# The Nitrobenzoxadiazole Derivative NBDHEX Behaves as *Plasmodium falciparum* Gametocyte Selective Inhibitor with Malaria Parasite Transmission Blocking Activity

**DOI:** 10.3390/ph15020168

**Published:** 2022-01-29

**Authors:** Giulia Siciliano, Veronica Di Paolo, Dante Rotili, Rossella Migale, Francesca Pedini, Marialuisa Casella, Serena Camerini, Daniele Dalzoppo, Rob Henderson, Tonnie Huijs, Koen J. Dechering, Antonello Mai, Anna Maria Caccuri, Marco Lalle, Luigi Quintieri, Pietro Alano

**Affiliations:** 1 Department of Infectious Diseases, Istituto Superiore di Sanità, 00161 Rome, Italy; giulia.siciliano@iss.it (G.S.); rossella.migale@gmail.com (R.M.); 2 Department of Pharmaceutical and Pharmacological Sciences, University of Padua, 35131 Padua, Italy; veronica.dipaolo@unipd.it (V.D.P.); daniele.dalzoppo@unipd.it (D.D.); 3 Department of Chemistry and Technology of Drugs, “Sapienza” University of Rome, 00185 Rome, Italy; dante.rotili@uniroma1.it (D.R.); antonello.mai@uniroma1.it (A.M.); 4 Department of Oncology and Molecular Medicine, Istituto Superiore di Sanità, 00161 Rome, Italy; francesca.pedini@iss.it; 5 Core Facilities, Istituto Superiore di Sanità, 00161 Rome, Italy; marialuisa.casella@iss.it (M.C.); serena.camerini@iss.it (S.C.); 6 TropIQ Health Sciences, 6534 AT Nijmegen, The Netherlands; r.henderson@tropiq.nl (R.H.); t.huijs@tropiq.nl (T.H.); k.dechering@tropiq.nl (K.J.D.); 7 Department of Chemical Sciences and Technologies, University of Tor Vergata, 00133 Rome, Italy; caccuri@uniroma2.it

**Keywords:** malaria, *Plasmodium falciparum* transmission, NBDHEX

## Abstract

This work describes the activity of 6-((7-nitrobenzo[*c*][1,2,5]oxadiazol-4-yl)thio)hexan-1-ol (NBDHEX) and of its newly identified carboxylic acid metabolite on the human malaria parasite *Plasmodium falciparum*. NBDHEX has been previously identified as a potent cytotoxic agent against murine and human cancer cells as well as towards the protozoan parasite *Giardia duodenalis*. We show here that NBDHEX is active in vitro against all blood stages of *P. falciparum*, with the rare feature of killing the parasite stages transmissible to mosquitoes, the gametocytes, with a 4-fold higher potency than that on the pathogenic asexual stages. This activity importantly translates into blocking parasite transmission through the *Anopheles* vector in mosquito experimental infections. A mass spectrometry analysis identified covalent NBDHEX modifications in specific cysteine residues of five gametocyte proteins, possibly associated with its antiparasitic effect. The carboxylic acid metabolite of NBDHEX retains the gametocyte preferential inhibitory activity of the parent compound, making this novel *P. falciparum* transmission-blocking chemotype at least as a new tool to uncover biological processes targetable by gametocyte selective drugs. Both NBDHEX and its carboxylic acid metabolite show very limited in vitro cytotoxicity on VERO cells. This result and previous evidence that NBDHEX shows an excellent in vivo safety profile in mice and is orally active against human cancer xenografts make these molecules potential starting points to develop new *P. falciparum* transmission-blocking agents, enriching the repertoire of drugs needed to eliminate malaria.

## 1. Introduction

**Figure 1:**
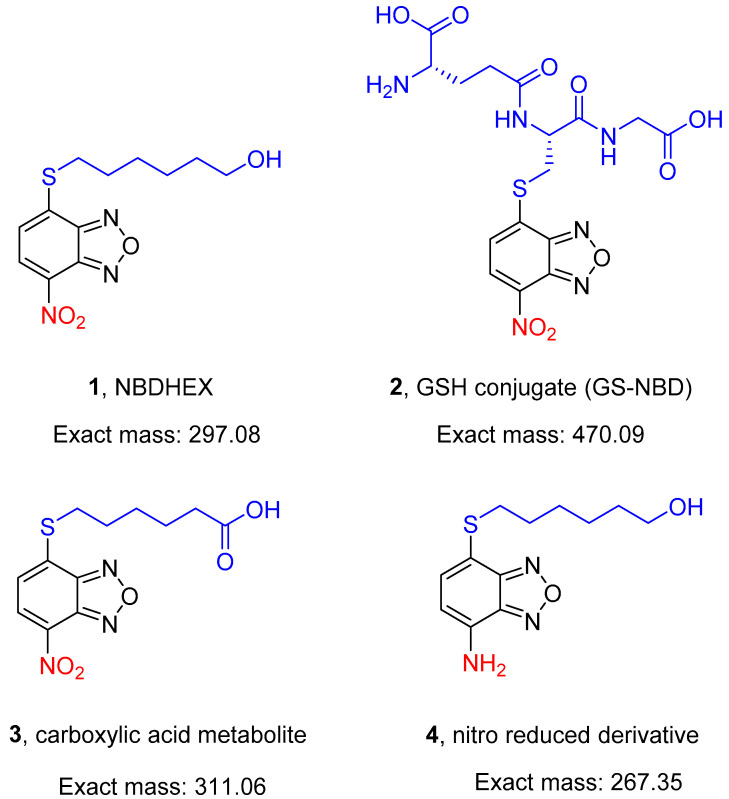
Structures of the compounds (**1**–**4**) evaluated in the present study.

A search for non-GSH-peptidomimetic inhibitors of human glutathione *S*-transferase P1-1 (GSTP1-1) for cancer therapy led to the discovery of 6-((7-nitrobenzo[*c*][1,2,5]oxadiazol-4-yl)thio)hexan-1-ol (NBDHEX, **1,** Figure 1). This nitrobenzoxadiazole (NBD) derivative is a potent mechanism-based inhibitor of both human GSTP1-1 and GSTM2-2 and exhibits remarkable cytotoxicity towards various cultured cancer cell lines (IC_50_ values in the low micromolar/submicromolar range) [1]. Notably, in vivo studies demonstrated that NBDHEX has an excellent safety profile in mouse models and is orally active in mice bearing human cancer xenografts [2,3]; **1** is also highly cytotoxic in vitro towards the trophozoite stage of the protozoan parasite *Giardia duodenalis*, an activity related to its ability to inhibit parasite FAD-dependent glycerol 3-phosphate dehydrogenase and thioredoxin reductase [4,5,6]. This evidence paves the way to explore the potential of **1** in the treatment of other protozoan parasite infections.

An early exploratory pharmacokinetic study in healthy mice receiving an intravenous bolus injection of **1** demonstrated a rapid decline in compound plasma levels, falling below the detection limit within 60 min (Caccuri, unpublished data). This finding may indicate a rapid distribution and/or elimination (by excretion and/or metabolism) of **1**. The metabolic instability of **1** was confirmed by recent in vitro data showing that it undergoes rapid non-enzymatic metabolism by reacting with GSH under physiological conditions of pH and temperature, giving rise to glutathionyl nitrobenzoxadiazole (GS-NBD, **2**, Figure 1) [7,8]. Moreover, a preliminary liver microsomal metabolic stability screen demonstrated that **1** undergoes metabolic elimination catalyzed by reduced nicotinamide adenine dinucleotide phosphate (NADPH)-supplemented liver microsomes from various animal species, including humans (Di Paolo, unpublished data).

The encouraging success achieved from 2000 to 2015 in lowering the burden of malaria, the disease caused by the protozoan parasite *Plasmodium*, is presently stalling, and the last WHO World Malaria Report indicates that the figures of 240 million cases and over 560,000 deaths annually have been fairly constant in the past 4–5 years [9]. The recent report of the first cases of failure of artemisinin-based combination therapy in Africa opens a dramatic scenario for the continent most hit by the most severe form of malaria, caused by *Plasmodium falciparum*, and highlights the urgent need for the discovery of novel drugs [10]. In the present frame of the effort to eliminate malaria, these will be needed to cure the infection by clearing asexual pathogenic parasite stages, whereas significant steps towards malaria elimination will need drugs able to block the transmission of the parasites from infected humans to the *Anopheles* mosquitoes. This key step of the *P. falciparum* life cycle is ensured by the *Plasmodium* gametocytes, the parasite stages developing in the infected host for about ten days through five stages (from I to V) of maturation. Gametocytes have different susceptibility to drugs compared to the asexual stages, with the mature stage V being the least sensitive to drug treatment [11,12,13].

Here, **1** and its in vitro-identified metabolites, namely the GSH conjugate **2** [7,8] and the carboxylic acid compound **3**, as well as the 7-amino derivative **4** (Figure 1), have been tested for the ability to kill the pathogenic asexual stages and the immature and mature transmissible stages of *P. falciparum*.

This work revealed that **1** can block parasite transmission in *Anopheles* mosquitoes and that both **1** and its oxidative metabolite **3** are preferentially active on gametocytes, particularly the mature stage V. Moreover, covalent adducts of **1**, but not of **4,** with specific cysteine residues have been reproducibly identified by mass spectrometry in five gametocyte proteins. These results make the NBD derivatives **1** and **3** important tools to uncover biological processes targetable by gametocyte-selective drugs and represent novel starting points to develop new *P. falciparum* transmission-blocking drugs.

## 2. Results

### 2.1. Compound ***1*** Has Gametocyte-Selective Transmission-Blocking Activity in P. falciparum

The activity of **1** was measured on different stages of *P. falciparum*, namely asynchronous asexual stages, immature (stage II/III), and mature (stage V) gametocytes. Wild-type asexual stages and gametocytes from the reference line 3D7 were tested using the pLDH enzymatic assay [14]. Transgenic parasites producing luciferase-expressing gametocytes were also used relying on a cell-based luciferase assay [15].

Assays measured the effect of **1** after a 48 h incubation. This was followed, as requested in the case of the pLDH assay, by a further 72 h incubation in a compound-free medium, whereas this step is not needed in the luciferase assays. The assays with transgenic immature gametocytes were conducted using the *P. falciparum* line NF54-*pfs16*-GFP/Luc line [11], and those with mature gametocytes used the line NF54*cg6*-*ULG8*CBG99 [13]. Results are shown in Table 1.

Independent luciferase reporter and pLDH enzymatic assays showed that **1** has nanomolar IC_50_ values on the viability of immature and mature gametocytes while it was virtually inactive on asexual stages.

The possibility that inhibitory activity of **1** on mature gametocytes leads to a blockade of parasite transmission through the *Anopheles* vector was tested in mosquito experimental infections with *P. falciparum* gametocytes from the NF54-*hsp70*-luc line and *Anopheles stephensi* by standard membrane-feeding assay (SMFA).

**Figure 2:**
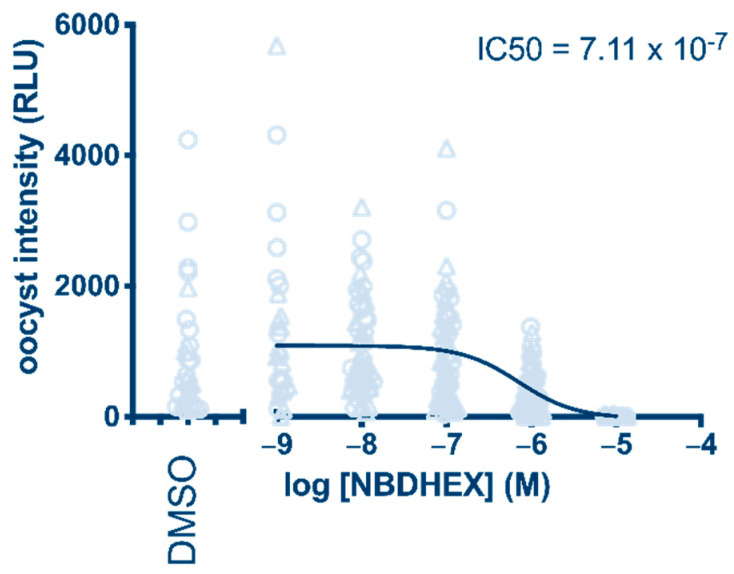
Determination of NBDHEX (**1**) transmission-blocking activity after 24 h treatment of *P. falciparum* gametocytes. Oocyst intensity is measured as relative light units (RLU) produced by the NF54-*hsp70*-luc parasite line.

The results proved that **1** has parasite transmission-blocking activity, showing a 0.7 µM IC_50_ value (oocyst intensity) after a 24 h treatment (Figure 2). In comparison, 10 µM dihydroartemisinin (DHA), which was taken along as a positive control, blocked oocyst formation by 83% (Supplementary Figure S1), in line with its activity reported previously [16].

### 2.2. Identification of ***3*** as a Product of the Oxidative Metabolism of ***1*** by Human Liver Microsomal and Cytosolic Fractions

Based on the results of a preliminary liver microsomal stability assay (see Introduction), exploratory in vitro drug metabolism experiments were conducted to get information on the nature of the liver microsomal metabolite(s) of compound **1** and, subsequently, to explore the possible biotransformation of the drug by NAD-dependent liver cytosolic oxidative enzymes [17].

**Figure 3:**
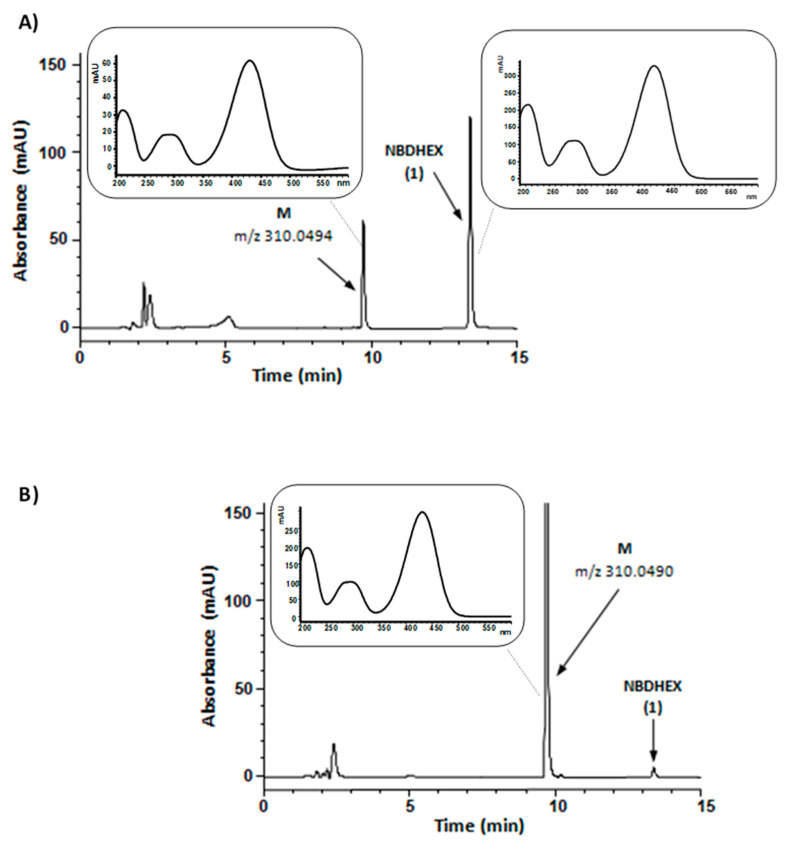
Representative HPLC chromatograms of reaction mixtures from a 60 min incubation (37 °C) of NADPH-supplemented human liver microsomes (panel **A**) or NAD-supplemented human liver cytosol (panel **B**) with 50 µM NBDHEX (**1**); in both mixtures, cofactor concentration was 0.5 mM. The samples were processed and analyzed by HPLC-DAD as described in Materials and Methods. In panel **A** chromatogram, percent areas of peaks M and NBDHEX (in terms of total peak area of the chromatogram) were 23 and 50, respectively; in panel **B** chromatogram, percent areas of peaks M and NBDHEX were 86 and 1, respectively. Panel A inset, UV-visible spectra of peak M and peak NBDHEX in the liver microsomes-containing mixture chromatogram. Panel B inset, UV-visible spectrum of peak M in the liver cytosol-containing mixture chromatogram. The reported *m*/*z* values were obtained from the ESI-mass spectra of collected peaks shown in Supplementary Figure S2 and refer to [M − H]^−^ ion.

In the first set of experiments, compound **1** was aerobically incubated with human liver microsomes and NADPH, an essential cofactor for microsomal oxidative reactions catalyzed by flavin monooxygenases and cytochrome P450, the latter one being the major enzyme system involved in drug metabolism [17]. The HPLC traces of the incubation mixtures demonstrated a new major absorption peak (peak M) whose retention time, UV-visible spectrum, and *m*/*z* value corresponded to that of synthetic compound **3**, i.e., the carboxylic acid resulting from the oxidation of the primary alcohol moiety of compound **1.** Both compound **1** and compound **3** UV-visible spectra were characterized by two absorption bands centered at 290 and 430 nm. A typical HPLC profile is shown in Figure 3, panel A; the negative mode ESI-MS spectrum of chromatographic peak M is shown in panel A of Supplementary Figure S2.

This finding is consistent with our previous evidence showing compound **1** instability after aerobic incubation with human liver microsomes and NADPH.

In the second set of experiments, compound **1** was aerobically incubated with human liver cytosol in the presence of NAD. Even under these conditions, compound **1** was converted into a metabolite (M) having retention time, UV-visible spectrum, and *m*/*z* value corresponding to that of synthetic compound **3** (Figure 3, panel B, and panel B of Supplementary Figure S2). The observed conversion of compound **1** into **3** by NAD-supplemented liver cytosol might conceivably result from the sequential activity of alcohol and aldehyde dehydrogenases, both of which have been found to be involved in the cytosolic oxidation of various primary alcohols into the corresponding carboxylic acids [17].

### 2.3. The Oxidative Metabolite of ***1*** (***3***) Retains Gametocyte Selective Inhibitory Activity

In a further set of experiments, the in vitro-identified metabolites of **1**, namely **2** and **3,** were analyzed for their activity on *P. falciparum* asexual and sexual stages.

Assays on asynchronous asexual parasites and on immature and mature gametocytes showed that the glutathione conjugate **2** was inactive on all parasite stages tested (Table 1, Supplementary Figure S3). Consequently, this compound was no longer tested.

To provide a more accurate comparison of the different compound activity on asexual stages, immature and mature gametocytes, a novel *P. falciparum* transgenic line, NF54 *hsp86*-PpyRE13 Luc (manuscript in preparation), was used in this set of experiments. In this parasite line, the expression of the PpyRE13 luciferase is driven by the constitutive promoter of the parasite *hsp86* gene, resulting in all parasite stages expressing the bioluminescent enzyme. This feature was thus exploited to measure parasite viability in asexual stages and gametocytes with the same assay and a homogeneous readout.

To validate the use of this parasite line, IC_50_ values for methylene blue (MB), a compound known to be effective against different blood stages of *P. falciparum* [11,13], were calculated from concentration–response curves with asexual stages and immature (stage II/III) and mature (stage V) gametocytes. Results (Table 1) were in line with IC_50_ values determined previously for MB (e.g., [11,13]).

In a preliminary experiment, the activity of **1** was tested on the different parasite stages of the NF54*-hsp86*-PpyRE13 line. Results confirmed the observation that **1** preferentially kills sexual stages, showing here an about 4-fold higher inhibition of mature gametocyte viability compared to asexual stages (Table 1).

The activity of metabolite **3** was compared to that of the parent **1** in a 48 h treatment on asexual stages and on immature (stage II/III) and mature (stage V) gametocytes of the same line. Results showed that **3** retained an inhibitory activity comparable to that of **1**, and importantly, like its parental compound, metabolite **3** showed a 3.3-fold higher potency on mature gametocytes than on asexual stages, although this difference was not statistically significant (*p* < 0.05) (Table 1).

### 2.4. Compounds ***1*** and ***3*** Have Promising Parasite/Host Selective Activities

In view of the possible use of the NBD derivatives **1** and **3** as starting points (hit/lead compounds) for the development of *P. falciparum* transmission-blocking drugs, an analysis of their cytotoxicity towards mammalian cells was performed in vitro using the VERO cell line. IC_50_ values for **1** and its active metabolite **3** were determined after continuous exposure of the cells to each compound for 24 or 48 h, with the registered antimalarial drug chloroquine used as a reference control.

Results showed that only **1** retained a significant cytotoxic activity after 48 h of exposure, with an IC_50_ value of 7.97 ± 0.22 µM (Table 1). Using the mean IC_50_ of **1** from three independent determinations on mature gametocytes (Table 1), a host/mature gametocyte selectivity index of 8.7 can be calculated. Interestingly, the concentration–response curves reported in Supplementary Figure S4 showed that the cytotoxicity of **3** at both incubation times and of **1** at the 24 h incubation time were indistinguishable from those of the licensed drug chloroquine (Table 1). As the IC_50_ of **3** on VERO cells resulted in >10 μM, a similar calculation could not be performed to obtain a host/mature gametocyte selectivity index for **3**.

### 2.5. ***1*** Has Lower Inhibitory Potency for Parasite GST than for Human GSTP1-1

As **1** is active on human tumor cells by inhibiting GSTP1-1, this may suggest that GST could be a relevant target of **1** in *P. falciparum*. To investigate this hypothesis, the inhibitory activity of **1** towards recombinant human GSTP1-1 was compared to that against recombinant PfGST.

**Figure 4:**
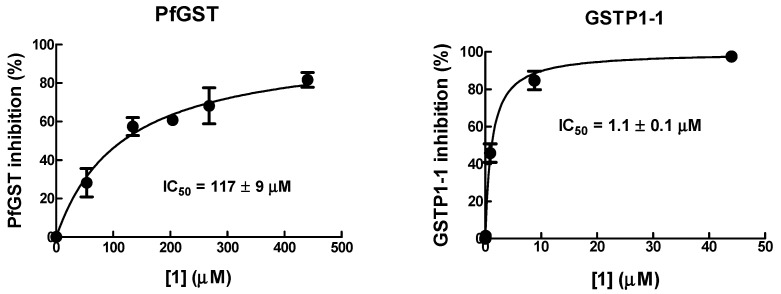
Inhibition of PfGST and human GSTP1-1 by **1**. The inhibitory potency of the **1** was determined by recording the activity of GST in the presence of increasing concentrations of **1**. IC_50_ values were determined as described in Materials and Methods.

The IC_50_ value of **1** towards PfGST was found to be ~100-fold higher than that towards human GSTP1-1 (Figure 4), suggesting that **1** has a relatively low affinity for parasite GST. Thus, PfGST is unlikely to be a relevant target of **1**.

### 2.6. Exposure to ***1*** but Not to Its Amine Analogue Results in the Formation of Adducts with Specific Cysteine Residues of P. falciparum Gametocyte Proteins

The activity of **1** towards tumor cells has been linked to its excellent electrophilic nature [1], and in *G. duodenalis*, cytotoxicity of **1** was associated with the formation of stable adducts with selected cysteine residues on specific parasite proteins, mainly with the 7-nitro moiety of **1** in the reduced form [4,5]. Furthermore, the 7-amino analog of **1** (**4** in Figure 1), which lacks the strong electron-withdrawing nitro group, was found to be not cytotoxic against *G. duodenalis* trophozoites in vitro (Lalle, unpublished results). Thus, **4** was used to investigate the relevance of the nitro moiety in mediating **1** cytotoxicity and the formation of an adduct between **1** and *P. falciparum* proteins.

Indeed, **4** did not show any cytotoxic activity either towards *P. falciparum* gametocytes or cultured VERO cells (Table 1).

Afterward, a mass spectrometry analysis was undertaken on gametocyte-infected and uninfected RBCs, treated with either **1** or **4**.

More than 500 proteins were identified in each sample, and mass spectra were inspected for the presence of peptides modified on cysteine or lysine residues by **1**. Results showed that only in infected RBC samples treated with **1** a small subset of *P. falciparum* proteins (Table 2; Supplementary Figure S5 and Supplementary Table S1) were consistently identified as modified on cysteine residues with a 265 Da mass increment compatible with **1** carrying an amine moiety instead of the nitro group at position 7 (Supplementary Figure S5). Noteworthy, no adduct could be identified in any of the samples derived from uninfected RBCs as well as in those with gametocyte-infected RBCs treated with either **4** or control DMSO, suggesting that the complete reduction of the 7-nitro group of **1** to its 7-amino counterpart does not likely precede the adduct formation with the cysteine residues.

Structural and sequence information of the five identified proteins modified by **1** was used to map the position of the cysteine residues carrying the **1** adduct. In the solved structure (2B4T) [18] of the parasite glyceraldehyde-3-phosphate dehydrogenase (PfGAPDH; PF3D7_1462800), the modified Cys58 locates on the enzyme surface, away from the catalytic site (Supplementary Figure S6). In the structure model (AF-C0H4V6-F1) of Pf14-3-3I protein (PF3D7_0818200) [19,20,21], the modified Cys149 resides in loop 5, encompassing alpha helices 5 and 6 [22], at one edge of the phosphorylated target binding groove (Supplementary Figure S7). In our automated molecular model of PfCDC48 (PF3D7_0619400), the Cys98 modified by **1** resides in the *N*-terminal domain, buried in the interface between the N- and the first ATPase domain, D1 [23,24] (Supplementary Figure S8). In our automated molecular model of PfTub-alpha2 (PF3D7_0422300), the modified Cys347 is located at the interface between α- and β-tubulin monomers (Supplementary Figure S9). Finally, it was impossible to map the Cys19 modification in the PfeL8 subunit of the 60S ribosomal complex (PF3D7_1424400), as it was impossible to model the structure of the first 40 residues as missing in available crystallographic determined structure [25,26].

## 3. Discussion

This work identified a novel chemotype able to block *P. falciparum* transmission through the *Anopheles* mosquito vector. Moreover, this work showed that oxidative liver metabolism of **1** gives rise to a metabolite, the carboxylic acid **3**, which retains antimalarial activity. Both the parent drug **1** and metabolite **3** preferentially kill all the parasite transmissible stages, i.e., the immature and the mature gametocytes, with up to a 4-fold higher potency than that measured on the pathogenic asexual stages (Table 1). Furthermore, mass spectrometry analysis showed that **1** consistently modifies a small set of identifiable *P. falciparum* proteins by forming covalent adducts with specific cysteine residues, likely contributing to the observed antiparasitic effects.

The high-throughput screening campaigns of recent years, which tested altogether over 5 million compounds on *P. falciparum* asexual stages, led to assembling a “Malaria Box” of 400 molecules as starting points for new antimalarial drugs [27]. Very few of these turned out to be active also on gametocytes [28,29], which prompted specific efforts in screening for compounds active on the parasite sexual stages. Only about 300,000 compounds have been so far tested in a variety of comparatively more demanding cell-based gametocyte assays, and the number of identified chemotypes inhibiting the viability of immature and/or mature gametocytes and, in some cases, blocking parasite transmission to mosquitoes is so far limited [30,31]. An observation from this endeavor is that the majority of these molecules are active also on asexual stages, which suggests that, despite the differences in the biology of the proliferative asexual stages and of the non-dividing gametocytes, few biological processes may be targetable specifically in the latter parasite stages.

The parasite transmission-blocking activity of **1** and the preferential activity of **1** and **3** against mature gametocytes with respect to asexual stages make these compounds starting points for both the identification of these elusive and specific processes and for the development of gametocyte-selective transmission-blocking leads.

Our observations indicate that **1** is a poor inhibitor of the *P. falciparum* GST, thus supporting a previous report on the poor affinity of NBD-Cl for PfGST [32]. The exclusion of PfGST as the main target for **1** opens the way to investigate alternative mechanisms of gametocyte-killing activity. As previously demonstrated in *G. duodenalis* [4,6], mass spectrometry identification of parasite proteins covalently modified on cysteine residues by **1** provides a first attempt to unravel the impact of this molecule on parasite biological functions. In our experimental condition, five gametocyte proteins were reproducibly identified being modified at specific cysteine residues by **1,** suggesting that these molecules might exert a pleiotropic effect on processes that are vital for the parasite. Although further experiments are needed to evaluate the impact of these modifications on the respective protein activities and the consequences on gametocyte viability, mapping the position of the modified cysteine residues through protein structural information may provide cues for speculation. In the parasite glycolytic enzyme glyceraldehyde-3-phosphate dehydrogenase (PfGAPDH), the modified Cys58 may allosterically alter the enzyme function, as observed for the mouse GAPDH following oxidative stress modifications of amino acid residues outside the enzyme active site [33]. The Pf14-3-3I belongs to a family of eukaryotic dimeric adaptor proteins binding to hundreds of proteins on specific phosphoserine/phosphothreonine binding motifs, thus being involved in the regulation of multiple key biological processes [34]. In the Pf14-3-3I identified here, one of the two *P. falciparum* structurally conserved isoforms [19,20,21], the modified Cys149 resides in loop 5, encompassing alpha helices 5 and 6 [22], a protein portion shown to contribute to structural diversity between plant 14-3-3 subgroups and therefore possibly involved in specificity for Pf14-3-3/target binding [35]. In PfCDC48, whose *P. berghei* orthologue inhibition affects the zygote to ookinete transition and blocks parasite transmission to mosquito [36], the localization of the modified Cys98 in the interface between the N- and D1-domains could possibly lead to conformational changes [23,24], as suggested by the consequence of point mutations in this region of the human CDC48 [37]. Finally, the Cys347 modification in PfTub-alpha2, mapped at the oligomer interface between α- and β-tubulin [38], may be specifically relevant to the parasite sexual stages as PfTub-alpha2 is differentially produced in male and female mature gametocytes [39]. It is also worth mentioning that the corresponding cysteine of the *G. duodenalis* tubulin alpha chain is targeted by **1** and associated with microtubule alterations [5].

In view of elucidating whether and how modifications of the above proteins may explain the preferential activity of **1** and **3** on mature gametocytes with respect to asexual stages, it is noticeable that a comparative analysis of the proteomes of asexual trophozoites, of immature and of mature gametocytes revealed that, with the exception of the constitutive 60S ribosomal protein L7a, the remaining four proteins are enriched in mature gametocytes [40].

The comparative analysis of the above cysteine modifications showed that these were detectable only in gametocyte-infected RBC treated with **1** and not with **4**, suggesting that the complete reduction of the 7-nitro group of **1** to its 7-amino counterpart likely takes place only after the previous adduct formation step(s). Recent observations that NBD derivatives are substrates of NADPH-dependent *P. falciparum* oxidoreductases led to propose a role for these molecules as “redox cyclers”, i.e., compounds depleting the cell cytosol reducing power [41]. This may lead to speculate that gametocyte enzymes, yet to be identified, are likely responsible for the nitroreduction process that seems to be involved in the generation/stabilization of the adducts between **1** and cysteine residues. In this respect, it is noticeable that mature gametocytes, parasite stages generally refractory to antimalarial drugs [11,12], have been shown to be exquisitely sensitive to redox unbalancing compounds and to decreases in parasite cell NADPH concentrations [13].

The parasite stage preferential activity of **1** and **3**, coupled with their low cytotoxicity towards cultured VERO cells, as an in vitro model of the host cell, is promising in view of the development of transmission-blocking gametocyte-selective drugs. Interestingly, the in vitro toxicity data presented in this work are consistent with the good safety profile of **1** and its phosphate ester derivative in tumor-bearing mice [42,43]. Furthermore, metabolite **3** showed toxicity towards cultured VERO cells about 5-fold lower than that of its parent **1** and comparable to that of the antimalarial drug chloroquine (Table 1), indicating that **3** may represent a better new antimalarial lead than **1**.

In the development of transmission-blocking drugs, dual active molecules able to kill both asexual stages and gametocytes may appear as ideal and are currently prioritized by several research groups and key drug discovery initiatives. However, one drawback is that such compounds are generally more potent on asexual stages than on mature gametocytes, which will likely lead to adopting treatment dosages suited to kill the former pathogenic stages but possibly suboptimal to clear the transmissible stages. In addition, dual active drugs are exposed to the risk of selecting resistance amongst the exposed, proliferating asexual parasites, likely to also abolish efficacy on the sexual stages [44]. Conceiving the development of drugs selectively targeting gametocytes and elucidating the underlying biological mechanisms and targets are therefore essential activities to enrich the current set of tools and strategies to eliminate malaria and, specifically, to contrast parasite transmission.

## 4. Materials and Methods

### 4.1. Chemicals and Synthesis of Compounds ***1***–***4***

Unless specified otherwise, chemicals were obtained from Merk Life Science S.r.l., Milan, Italy.

**Figure 5:**
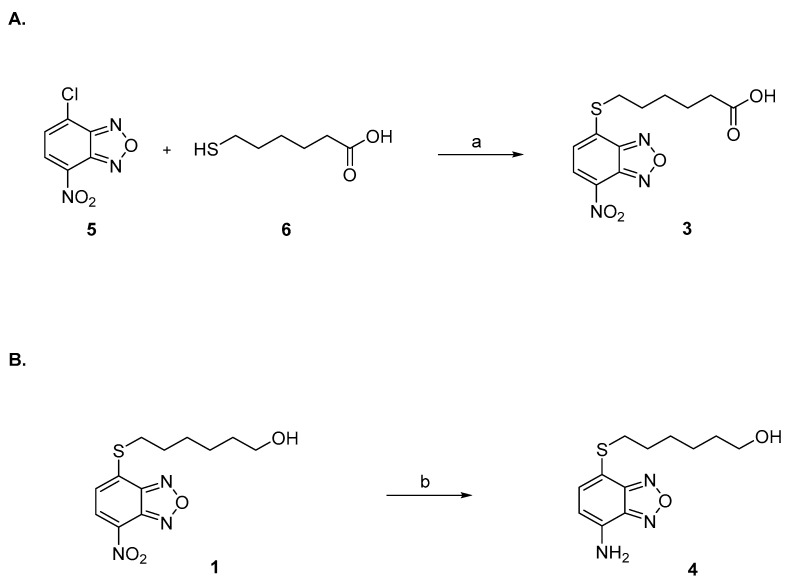
(**A**) Reagents and conditions: (a) NaHCO3 0.5 M, THF, r.t. (**B**) Reagents and conditions: (b) Fe powder, HCl 12 N, DCM, MeOH, r.t.

Compounds **1** and **2** were synthesized as previously described [1,45]. Compound **3** was prepared by reaction under mild basic conditions between the commercially available 4-chloro-7-nitrobenzo[*c*][1,2,5]oxadiazole (**5**) and 6-mercaptohexanoic acid (**6**) (Figure 5A). Compound **4** was synthesized by reduction of **1** with iron powder and concentrated hydrochloric acid (Figure 5B).

Preparation of compound **3**. A solution of 4-chloro-7-nitrobenzo[*c*][1,2,5]oxadiazole (**5**) (19 mg, 95.2 μmol, 1.05 eq.) in THF (200 μL) was added in two portions at room temperature to a solution of 6-mercaptohexanoic acid (**6**) (13.5 mg, 91.1 μmol, 1.0 eq.) in 0.5 M NaHCO_3_ (2 mL). After 45 min of vigorous stirring, the reaction mixture was quenched with ethyl acetate (2 mL), and the organic phase was then discarded. After acidification with 6 M HCl (1 mL), the aqueous phase was extracted with ethyl acetate (4 × 2 mL). The combined organic phases were then evaporated almost completely with a light stream of nitrogen. The resulting residue was dissolved with a mixture of water (1 mL) and ethanol (0.5 mL), and the obtained solution was placed in the freezer at −18 °C to promote the crystallization of the desired compound **3** (Figure 5A). ^1^H-NMR (DMSO) δ 1.48 (m, 2H, -SCH_2_CH_2_C*H*_2_CH_2_CH_2_COOH), 1.58–1.65 (m, 4H, -SCH_2_C*H*_2_CH_2_C*H*_2_CH_2_COOH), 2.31–2.38 (t, 2H, -SCH_2_CH_2_CH_2_CH_2_C*H*_2_COOH), 2.98–3.03 (t, 2H, -SC*H*_2_CH_2_CH_2_CH_2_CH_2_COOH), 7.54 (d, 1H, C*H* benzoxadiazole ring), 8.26 (d, 1H, C*H* benzoxadiazole ring), 12.15 (br s, 1H, COO*H*). MS (ESI), *m/z*: 310.1 [M − H]^−^.

Preparation of compound **4**. Concentrated (12 N) hydrochloric acid (0.8 mL) and iron powder (75 mg, 4 eq.) were added to a solution of **1** (100 mg, 0.34 mmol, 1 eq.) in DCM (12 mL) and MeOH (6 mL). After stirring at room temperature for 6 h, the reaction mixture was quenched with water (30 mL) and extracted with ethyl acetate (4 × 15 mL). The combined organic phase was then dried over sodium sulphate and evaporated under reduced pressure. The resulting crude was purified by silica gel column chromatography eluting with a mixture of ethyl acetate: petroleum ether (5 to 50%) and provided **4** as an orange powder (Figure 5B). ^1^H-NMR (CDCl_3_) δ 1.25 (br s, 1H, -SCH_2_CH_2_C*H*_2_CH_2_ CH_2_CH_2_O*H*), 1.28–1.66 (m, 8H, -SCH_2_C*H*_2_C*H*_2_C*H*_2_C*H*_2_CH_2_OH), 3.01 (t, 2H, -SC*H*_2_CH_2_CH_2_CH_2_CH_2_CH_2_OH), 3.62 (br t, 2H, -SCH_2_CH_2_CH_2_CH_2_CH_2_C*H*_2_OH), 4.60 (br, s, N*H*_2_), 6.30 (d, 1H, C*H* benzoxadiazole ring), 7.22 (d, 1H, C*H* benzoxadiazole ring). MS (ESI), *m/z*: 268.1 [M + H]^+^.

The ^1^H and ^13^C-NMR spectra of all synthesized compounds and their relative mass spectra (ESI-MS) have been determined and are shown in the Supplemental file ^1^H-NMR_^13^C-NMR_ESI-MS spectra of compounds **1**–**4**.

### 4.2. In Vitro Drug Metabolism Studies

#### 4.2.1. Metabolite Profiling of Compound **1** in Human Liver Microsomes and Human Liver Cytosol

The incubation mixtures (final volume, 0.2 mL) consisted of 50 mM KH_2_PO_4_ (pH 7.4), compound **1** (final concentration, 10 or 50 µM), 0.5 mg of protein/mL of pooled human liver microsomes (Xenotech LLC, Lenexa, KS, USA) or pooled human liver cytosol (Xenotech LLC), and 0.5 mM NADPH (mixtures containing liver microsomes) or oxidized nicotinamide adenine dinucleotide (NAD; mixtures containing liver cytosol). The reactions were started following a 3 min thermal equilibration at 37 °C by adding the microsomes or the cytosolic fraction, conducted at 37 °C under aerobic conditions for different time periods, and terminated by adding 0.1 mL of ice-cold acetonitrile. After protein removal by centrifugation at 20,000× *g* for 10 min (4 °C), an aliquot of the supernatant was analyzed by HPLC with diode array detection, and metabolite peaks were collected manually and subjected to mass spectrometry as described below.

#### 4.2.2. HPLC-Diode Array Detection (DAD) Analysis

Analysis was conducted using a Hewlett-Packard series 1100 HPLC system Agilent Technologies Inc., formerly Hewlett-Packard Co., Palo Alto, CA, USA) equipped with a degasser, a quaternary pump, an autosampler, a column oven, and a diode-array detector; data were collected and integrated using the Agilent ChemStation software. Chromatographic conditions were as follows: column, Agilent Zorbax Eclipse XDB-C18 (3.0 × 150 mm, 5 μm; Agilent Technologies Inc.); mobile phase, 10 mM ammonium bicarbonate, pH 6.8/acetonitrile (90:10 *v*/*v*; solvent A) and acetonitrile (solvent B); elution program, isocratic elution with 100% solvent A for 2 min, linear gradient from 0 to 70% solvent B in 8 min, followed by isocratic elution with 70% solvent B for 5 min; post-run time, 7 min; flow rate, 0.4 mL/min; injection volume, 30 μL; column temperature, 28 °C; the diode-array detector was set to monitor at 433 nm, and the online UV-visible spectra were recorded in the scanning range of 200–600 nm. Under the above conditions, the retention times of compound **1** and authentic compound **3** were 13.4 and 9.7 min, respectively.

#### 4.2.3. Mass Spectrometry Analysis

HPLC fractions corresponding to the “M” peaks (see Results) were collected and subjected to electrospray ionization time-of-flight mass spectrometry (ESI-TOF-MS) using a Xevo G2-S QTof (Waters Corp., Milford, MA, USA). The mass spectrometer operated under the following conditions: electrospray in negative ion mode (ES-); source temperature, 100 °C; desolvation gas temperature, 350 °C; capillary voltage, 2.5 kV; sampling cone voltage, 15 V; collision energy, 6 V. Data were collected by full scan mode (scan range of *m*/*z*, 50–1200) and evaluated by the MassLynx software (Waters Corp.).

### 4.3. GST Inhibition Experiments

The 6xHis-tagged PfGST expression plasmid was a kind gift of Prof. K. Becker (Justus Liebig University Giessen). Recombinant GSTP1-1 and *P. falciparum* PfGST were expressed in *Escherichia coli* and purified as previously described [46,47]. Protein concentration was calculated assuming an ε 1 mg/mL of 1.30 and 1.1 at 280 nm for GSTP1-1 and PfGST, respectively. Before kinetics analysis, the purified PfGST was incubated with 5 mM GSH to trigger the transition from a low affinity towards a high-affinity conformation [32].

PfGST and GSTP1-1 catalytic activities were followed spectrophotometrically at 340 nm, where the reaction product absorbs (ε = 9.6 mM^−1^ cm^−1^) [48]. The assay mixture contained 1 mM GSH and 1 mM 1-chloro-2,4-dinitrobenzene (CDNB), as cosubstrate, in 1 mL of 0.1 M potassium phosphate buffer, pH 6.5. The rate of increase in absorbance at 340 nm was monitored at 25 °C after the addition of the appropriate amount of enzyme. The inhibitory potency of compound **1** was determined by recording the activity of GST in the presence of increasing concentrations of **1**. The IC_50_ values (concentration of inhibitor resulting in 50% enzyme inhibition) were determined by non-linear regression curve fit analysis using GraphPad Prism 6.0 (GraphPad Software, La Jolla, CA, USA).

### 4.4. Parasite Lines and Culture Protocols

Parasite lines used in this work were the reference wild-type clone 3D7A [49], from dr. Richard Carter, University of Edinburgh, UK; line NF54 *pfs16*-GFP-PyLUC [11], from dr. David Fidock, Columbia University, New York, USA; line NF54cg6-*ULG8*CBG99 [13], produced in the authors’ laboratory at Istituto Superiore di Sanità; line NF54-*hsp70*-luc [50], produced in the authors’ laboratory at TropIQ Health Sciences, and line NF54 *hsp86*-PpyRE13, produced in the authors’ laboratory at Istituto Superiore di Sanità (manuscript in preparation).

*P. falciparum* asexual parasites were cultured in type 0+ human erythrocytes at 5% hematocrit in RPMI 1640 (Life Technologies, Paiseley, UK). The medium was supplemented with 10% heat-inactivated O+ human serum (IBBI, Memphis, TN, USA) and 0.36 mM hypoxanthine at 37 °C in a modified atmosphere (3% O_2_, 4% CO_2_, and N_2_) by established methods [51]. Gametocyte production was induced by seeding asexual parasite cultures at 0.1% parasitemia and 5% hematocrit with no further addition of uninfected red blood cells. At the appearance of the oat-shaped stage I gametocytes, 50 mM N-Acetyl Glucosamine (NAG) was added for 3 days to clear residual asexual parasites [52], followed by additional 5 days of culturing in the absence of NAG.

### 4.5. P. falciparum Drug Sensitivity Assays

Cell-based assays on asexual and sexual parasite stages measuring the activity of the parasite pLDH enzyme are described in detail in [14].

The assays measuring the activity of the luciferase reporter genes are described in detail in [15]. In brief, to calculate IC_50_ values, compounds **1**, **2**, **3**, and **4** were serially diluted across ten two-fold dilutions and dispensed in 96-well plates in a final volume of 100 µL/well. Asexual stage parasites at 1,5% parasitemia and synchronous 8 × 10^4^ immature (stage II/III) and mature (stage V) gametocytes were resuspended in 100 µL of complete medium and incubated with the compounds at 37 °C for the time indicated. Cell viability was evaluated by adding a non-lysing formulation of 0.5 mM D-Luciferin substrate [15] and measuring luciferase activity for 1 s on a Varioskan™ Flash Multimode Reader (Thermo Scientific, Waltham, MA, USA). The percent viability was calculated as a function of drug concentration. Curve fitting was obtained by non-linear regression analysis (GraphPad Prism 6.0).

### 4.6. Standard Membrane Feeding Assay (SMFA)

Compound **1** was diluted in DMSO and then in RPMI medium (Gibco, 51800043, Life Technologies) to achieve a final DMSO concentration of 0.1%. Diluted samples were combined with stage V gametocytes from *P. falciparum* strain NF54-*hsp70*-luc and incubated for 24 h. Subsequently, human red blood cells and human serum with the sample were added to achieve a hematocrit of 50%. The blood meal was fed to two-day-old *Anopheles stephensi* mosquitoes that were starved the night prior to the blood meal. Mosquito infection was analyzed 8 days post-feeding by luminescence measurements of whole mosquitoes as described previously [48]. For each condition, luminescence intensity was analyzed in mosquitoes from two replicate feeders with 15–24 mosquitoes per feeder (N = 2, *n* = 15–24). Luminescence background levels were determined from 15–24 uninfected mosquitoes. Controls: vehicle (0.1% DMSO) in duplicate.

### 4.7. Cytotoxicity Assay

For cytotoxicity assay, commercial Vero cells (*Cercopithecus Aethiops*—African green monkey kidney cells, European Directorate for the Quality of Medicines & HealthCare code V0180000) were plated at a density of 5 × 10^2^ per well in 96-well plates in RPMI complete medium containing 10% FBS (Gibco, Life Technologies, Paiseley, UK) at 37 °C in 5% CO_2_. After 48 h, compounds were individually added to the culture medium at different concentrations (0.075, 0.15, 0.3, 0.65, 1.25, 2.5, 5, and 10 µM), and cells were further incubated for either 24 h or 48 h. For each drug and concentration, measurements were performed in triplicate. Cell viability was evaluated after 24 h and 48 h using the CellTiter-Blue Viability Assay (Promega Inc., Madison, WI, USA) following the manufacturer’s instruction. Briefly, 20 μL of the CellTiter-Blue^®^ Reagent was added directly to each well, the plates were incubated for 2 h at 37 °C to allow viable cells to reduce the indicator dye resazurin into the highly fluorescent resorufin, and the fluorescent signal was measured by MultiLabel Plate Reader VICTOR X3™ (Perkin Elmer, Waltham, MA, USA). The IC_50_ values were determined by non-linear regression curve fit analysis using GraphPad Prism 6.0 (GraphPad Software, La Jolla, CA, USA).

### 4.8. Protein Mass Spectrometry Analysis

Approximatively 1 × 10^7^ purified stage III-IV gametocyte-infected RBCs were incubated at 37 °C with DMSO or with 100 µM of either compound **1** or compound **4** for 2 h. As control, 1 × 10^7^ uninfected RBCs were treated in parallel. Following incubation, RBCs or gametocyte-infected RBCs were collected and washed twice with PBS, pelleted, and frozen at −80 °C until use. For protein extraction, RBCs or gametocyte-infected RBC pellets were resuspended in 50 µL of PBS/Triton-X100 1% supplemented with protease/phosphatase inhibitors (Halt™ Protease and Phosphatase Inhibitor Single-Use Cocktail, ThermoFisher Scientific) to approximately 2 × 10^5^ cells/µL, lysed by incubation on ice for 1 h and the supernatant collected following centrifugation at 13,000 rpm for 15 min at 4 °C. The insoluble pellets were resuspended in 30 µL Laemlie buffer (BioRad) and solubilized by incubation at 75 °C for 10 min. Proteins from the supernatants and insoluble pellets were separated on a NuPAGE 4–12% (InvitrogenTM, ThermoFisher Scientific, Waltham, MA, USA) in MOPS buffer. The gel was stained with Colloidal Blue Staining Kit (Invitrogen), and gel slices containing protein bands in the range 250–30 KDa were excised and digested with trypsin (Promega Corporation, Madison, WI, USA), as previously described [53]. Nanoflow reversed-phase liquid chromatography–tandem mass spectrometry (RP-LC-MS/MS) analyses of peptide mixtures were performed on three independent biological replicates using an HPLC Ultimate 3000 (DIONEX, Sunnyvale, CA, USA) coupled with a linear ion trap (LTQ, Thermo Electron, San Jose, CA, USA) mass spectrometer equipped with a nano source, as described [5]. Briefly, peptides were desalted in a trap column and separated in a 10 cm long fused silica capillary (Silica Tips FS 360-75-8, New Objective) slurry packed in house with 5 μm, 200 Å pore size C18 resin (Michrom BioResources, Auburn, CA, USA). Mass spectra were acquired in positive mode (HV potential 1.7–1.8 kV), and MS/MS spectra were acquired in data-dependent mode with the top 5 most intense precursor ions selected for the fragmentation in each cycle time using 35% normalized collision ion energy. Data were analyzed matching the tandem mass spectra against the *P. falciparum* 3D7A protein database (PlasmoDB version 31) using the software Proteome Discoverer 1.4 (Thermo Electron). Precursor and fragment ions were searched with 2 and 0.8 Da tolerance, respectively. The following match parameters were taken into account: fully tryptic cleavage constraints (two miss-cleavage allowed), static cysteine carbamidomethylation, and variable methionine oxidation.

Putative adducts on cysteine or lysine residues with **1** were searched by taking into account **1** as intact or nitro-reduced.

Only proteins identified with at least 2 peptides were taken into account. The false discovery rate was set to 1% and determined by searching the reverse database using the Percolator node, based on q values. All the spectra relative to peptides modified by NBDHEX were also manually reviewed.

The mass spectrometry proteomics data have been deposited to the ProteomeXchange Consortium (http://proteomecentral.proteomexchange.org, accessed on 12 December 2021) via the PRIDE partner repository [54] with the dataset identifier PXD030336.

### 4.9. Bioinformatic Analysis

Multiple sequence analyses were performed with the MultAlin tool (http://multalin.toulouse.inra.fr/multalin/multalin.html, accessed on 3 November 2021) and manually refined. A highly reliable predicted model for Pf14-3-3I (PF3D7_0818200; model AF-C0H4V6-F1) was downloaded from the AlphaFold database (https://alphafold.ebi.ac.uk, accessed on 8 November 2021). Automated modeling for PfCDC48 (PF3D7_0619400) and PfTUB (PF3D7_1462800) were performed with SWISS-MODEL server (https://swissmodel.expasy.org, accessed on 9 November 2021) using as a model the cryo-EM structure of human p97 bound to ATPgS (Conformation II) (pdb: 5FTM) and the atomic structure monomeric tubulin alpha chain of *Toxoplasma gondii* (pdb: 7miz.39.A), respectively. Relevant model parameters were: Global Model Quality Estimate (GMQE) = 0.66 and QMEANDisCo global score = 0.67 ± 0.05, for PfCDC48 model, and GMQE = 0.84 and QMEANDisCo global score = 0.80 ± 0.05, for PfTUB. The crystal structure of PfGAPDH (2B4T) [17] was downloaded from RCSB Protein Data Bank (https://www.rcsb.org, accessed on 8 November 2021). UCSF Chimera ver.1.15 and ChimeraX [55,56] were used for visualization and analyses of experimentally determined structures and 3D models.

## 5. Conclusions

In conclusion, this work reveals that the nitro-oxadiazole compound NBDHEX, a potent cytotoxic agent against murine and human cancer cells as well as against the protozoan parasite *Giardia duodenalis*, is active in vitro against all blood stages of the human malaria parasite *Plasmodium falciparum* and, importantly, it is able to block parasite transmission through the Anopheles mosquito vector.

An attractive feature of NBDHEX and of its newly described carboxylic acid metabolite is that both are preferentially active on the *P. falciparum* mature gametocytes, the human-to-mosquito transmission stages, than on immature gametocytes and on the parasite asexual stages.

Treatment of *P. falciparum* gametocytes with NBDHEX, but not with an NBDHEX nitro-reduced derivative, results in the formation of adducts with specific cysteine residues in five parasite proteins, an observation paving the way to the elucidation of the compound mechanism of action.

NBDHEX and its carboxylic acid can thus contribute to uncovering biological processes targetable by gametocyte-selective drugs and represent starting points for the development of novel *P. falciparum* transmission-blocking drugs.

## Figures and Tables

**Table 1:** IC_50_ values (µM) of **1**, **2**, **3**, and **4** and Methylene blue (MB) on *P. falciparum* asexual and sexual stages measured in luciferase and pLDH assays. IC_50_ values (µM) of compounds **1**, **3**, **4**, and chloroquine (CQ) on Vero cells measured in CellTiter-Blue assays.

Compound	Assay Type (Hours of Treatment)	IC_50_ Values (µM)			
		Asexual Parasites	Gametocytes (Stage II/III)	Gametocytes (Stage V)	VERO Cells
**1**	pLDH (48 + 72 h)	>10 (*n* = 4) ^a^	0.5 ± 0.4 (*n* = 2) ^a^	0.3 ± 0.1 (*n* = 2) ^a^	
	Luciferase (24 h)		0.5 ± 0.2 (*n* = 2) ^b^	0.5 ± 0.1 (*n* = 4) ^c^	
	Luciferase (48 h)	7.9 ± 4.5 (*n* = 6) ^d^	6.9 ± 3.7 (*n* = 5) ^d^	1.9 ± 0.7 (*n* = 6) ^d^	
	CellTiter-Blue (24 h)				>10 (*n* = 2)
	CellTiter-Blue (48 h)				7.9 ± 0.2 (*n* = 2)
**2**	pLDH	>10 (*n* = 2) ^d^	>10 (*n* = 1) ^d^	>10 (*n* = 1) ^d^	
	Luciferase (24 h)		>10 (*n* = 1) ^d^	>10 (*n* = 1) ^d^	
	Luciferase (48 h)		>10 (*n* = 1) ^d^	>10 (*n* = 1) ^d^	
**3**	Luciferase (48 h)	16.4 ± 9.3(*n* = 2) ^d^	1.1 (*n* = 1) ^d^	5.0 ± 0.7 (*n* = 2) ^d^	
	CellTiter-Blue (24 h)				>10 (*n* = 2)
	CellTiter-Blue (48 h)				>10 (*n* = 2)
**4**	Luciferase (48 h)	5.2 ± 2.1 (*n* = 3) ^d^	>10 (*n* = 3) ^d^	>10 (*n* = 3) ^d^	
	CellTiter-Blue (24 h)				>10 (*n* = 2)
	CellTiter-Blue (48 h)				>10 (*n* = 2)
MB	Luciferase (48 h)	0.02 ± 0.06 (*n* = 2) ^d^	0.02 ± 0.07 (*n* = 2) ^d^	0.4 ± 0.3 (*n* = 2) ^d^	
CQ	CellTiter-Blue (24 h)				>10 (*n* = 2)
	CellTiter-Blue (48 h)				>10 (*n* = 2)

^a^ *P. falciparum* line 3D7, ^b^ *P.falciparum* line NF54 *pfs16*-GFP-PyLUC, ^c^ *P. falciparum* line NF54*cg6-ULG8*CBG99, ^d^ *P. falciparum* line NF54 *hsp86*-PpyRE13.

**Table 2:** *P. falciparum* gametocyte proteins with **1** adducts detected in at least 2 out of 3 biological replicates ^a^.

PlasmoDB Gene ID	Protein Description	Modified Peptide (Residues) ^b^	Modified Residue	Δm ^c^
PF3D7_1462800	Glyceraldehyde-3-phosphate dehydrogenase (GAPDH)	YDSVHGQFPCEVTHADGFLLIGEK (49–72)	Cys58	+265
PF3D7_0818200	14-3-3 isoform I (14-3-3I)	YISEFSCDEGK (143–153)	Cys149	+265
PF3D7_0619400	Cell division cycle protein 48 (Cdc48)	VCLGDVVYVK (97–106)	Cys98	+265
PF3D7_0422300	Alpha tubulin 2 (Tub-α2)	SIQFVDWCPTGFK (340–352)	Cys347	+265
PF3D7_1424400	60S ribosomal protein L7a (eL8)	TPAPCPLSTK (15–24)	Cys19	+265

^a^ Adducts detected in gametocyte protein lysate following exposure with 100 µM of **1** for 2 h (see Materials and Methods). ^b^ Modified cysteines are underlined. ^c^ Delta mass (Δm) is the deviation of the measured mass from the theoretical mass of the peptide.

## Data Availability

The mass spectrometry proteomics data have been deposited to the ProteomeXchange Consortium (http://proteomecentral.proteomexchange.org; accessed on 12 December 2021) via the PRIDE partner repository [51] with the dataset identifier PXD030336.

## Supplementary Materials

The following supporting information can be downloaded at: https://www.mdpi.com/article/10.3390/ph15020168/s1, Figure S1: Positive (10 µM Dihydroartemisinin) and negative (DMSO) controls in SMFA experiment; Figure S2: ESI-TOF-MS spectrum of peak “M” in the chromatogram of Figure 3A,B. Mass spectra peaks at *m*/*z* 195.98 represent a fragment ion arising from the loss of C6H10O2 (carboxyhexyl moiety) from the parention (*m/z* = 310.04); Figure S3: Representative concentration-response curves of the activity of 2 on asexual parasites, stage II/III and stage V gametocytes; Figure S4: Representative concentration-response curves of the cytotoxic activity of chloroquine, of 1 and of 3 on VERO cells; Figure S5: MS/MS spectra (panel a–e) of the modified peptides deriving from P. falciparum proteins GAPDH, 14-3-3I, Tubulin-α2, Cdc48, and 60S ribosomal L7-3, detected in the samples treated with 1. In each panel the sequence of the peptide is reported; c (in bold) indicates the modified cysteine carrying a mass increase of 265 Da corresponding to the adduct with 7-nitro form of 1 (the formula is reported in panel f). The b and y series fragment ions reported on each sequence are detected in each spectrum. The red and blue peaks in each spectrum correspond to the b and y series fragment ions, respectively; Figure S6: Localization of 1-modified Cys58 in the PfGAPDH structure. A) Two protomers of the tetrameric PfGAPDH structure (PDB: 2B4T) are shown as cartoon. Different domains are coloured in one of the protomer: N-terminal NAD binding domain (light blue); S-loop (yellow); C-terminal catalytic domain (magenta). NAD molecules are shown (red). Side chains of the two catalytic residues Cys 153 and His180 are shown by ball and stick representation (black). Side chain of Cys58 is shown by spheres (orange). Figure was created with Chimera 1.15 [55]. B) Multiple sequence alignment of Plasmodium falciparum PfGAPDH (Acc. N. Q8IKK7) with Homo sapiens GAPDH isoform 1 (HsGAPDH1, NP_002037.2), isoform 2 (HsGAPDH2, NP_001243728.1) and isoform 4 (HsGAPDH4, NP_001344872.1). Sequence are numbered according to PfGAPDH. Different domains are coloured: N-terminal NAD binding domain (light blue); S-loop (yellow); C-terminal catalytic domain (magenta). NAD molecules are shown (red). Catalytic residues Cys 153 and His180 of PfGAPDH are underlined and marked by hashtags. Cys58 is highlighted (red) and marked by asterisk. Gaps are indicated by dashes. Conserved residues in the alignment are indicated by dots; Figure S7: Localization of 1-modified Cys149 in the Pf14-3-3I modelled structure. A) Homology model of Pf14-3-3I (light blue) (model AF-C0H4V6-F1) downloaded from AlphaFold database (https://alphafold.ebi.ac.uk, accessed on 8 November 2021) is shown as cartoon. Alpha helices are marked (α1- α7). The side chains of the highly conserved residues (Lys135, Arg142, Leu187, Asn188, Val191 and Arg142) involved in phosphorylated target binding are shown by ball and stick representation (black). Side chain of Cys148 is shown by spheres (orange). Figure was created with ChimeraX [56]. B) Multiple sequence alignment of Plasmodium falciparum Pf14-3-3I (Acc. N. C0H4V6.1) with Homo sapiens 14-3-3 isoform beta (P31946.3, Hs14-3-3β), epsilon (P62258.1, Hs14-3-3ε), eta (Q04917.4, Hs14-3-3η), gamma (NP_036611.2; Hs14-3-3γ), sigma (P31947.1, Hs14-3-3σ) and theta (P27348.1, Hs14-3-3θ). Sequence are numbered according to Pf14-3-3I. Highly conserved residues (Lys135, Arg142, Leu187, Asn188, Val191 and Arg142) involved in phosphorylated target binding are in bold and highlighted (yellow). Cys149 is highlighted (red) and marked by asterisk. Gaps are indicated by dashes. Conserved residues in the alignment are indicated by dots; Figure S8: Localization of 1-modified Cys98 in the PfCdc48 modelled structure. A) Homology model of PfCdc48 was obtained using SwissModel (see Materials and Methods). A single protomer of the hexameric PfCdc48 is shown as cartoon. Different domains are marked and coloured: N-terminal domain (green); ATPase domain 1 (D1, magenta); ATPase domain 2 (D2, yellow). Loop between domain N and D1 (L1, dark blue), loop between domain D1 and D2 (L2, blue). Side chain of Cys98 is shown by spheres (orange). Figure was created with ChimeraX [56]. B) Multiple sequence alignment of Plasmodium falciparum PfCdc48 (Acc. N. XP_966179.2) with Homo sapiens Cdc48 isoform 1 (HsCDC48_1; NP_009057.1) and isoform 2 (HsCDC48_1; NP_001341856.1). Sequence are numbered according to PfCdc48. Different domains are coloured: N-terminal domain (green); ATPase domain 1 (D1, magenta); ATPase domain 2 (D2, yellow). Cys98 is highlighted (red) and marked by asterisk. Gaps are indicated by dashes. Conserved residues in the alignment are indicated by dots; Figure S9: Localization of 1-modified Cys98 in the PfTub-α2 modelled structure. A) Homology model of PfTub-α2 (blue) was obtained using SwissModel (see Materials and Methods), superimposed with Bos taurus α-tubulin in the α-tubulin (yellow)/β-tubulin (gray) oligomer structure (PDB ID: 4O2B), and visualized as cartoon. Tubulin subunits are indicated. Side chain of Cys347 is shown by spheres (orange). Figure was created with ChimeraX [56]. B) Multiple sequence alignment of Plasmodium falciparum PfTub-α2 (PfalphaTUB; Acc. N. Q8IFP3) with Homo sapiens α-tubulin (HsalphaTUB; gi|37492) and β-tubulin (HsbetaTUB; CAA56071.1), and Giardia duodenalis α-tubulin (GdalphaTUB; GL50803_103676) Sequence are numbered according to PfTub-α2. Residues at the interface between α-tubulin β-tubulin are red, residues that bind GTP/GDP are in light blue [39]. Cys347 is highlighted (red) and marked by asterisk. Gaps are indicated by dashes. Conserved residues in the alignment are indicated by dots; Table S1: List of P. falciparum proteins carring cysteine adduct with compound 1. Number of identified peptides for each experiment (Replica) and drug treatment is reported. “z” is the observed peptide charge, “Xcorr” is SEQUEST X-correlation, and “PEP” is the PSM associated posterior error probability.

## References

[1] Ricci G., De Maria F., Antonini G., Turella P., Bullo A., Stella L., Filomeni G., Federici G., Caccuri A.M. (2005). 7-Nitro-2,1,3-benzoxadiazole derivatives, a new class of suicide inhibitors for glutathione S-transferases. Mechanism of action of potential anticancer drugs. J. Biol. Chem..

[2] Dalzoppo D., Di Paolo V., Calderan L., Pasut G., Rosato A., Caccuri A.M., Quintieri L. (2017). Thiol-Activated Anticancer Agents: The State of the Art. Anticancer Agents Med. Chem..

[3] Sha H., Dong S., Yu C., Zou R., Zhu Y., Lu Y., Zhang J., Cao H., Chen D., Wu J. (2020). In Vitro and in Vivo Efficacy of NBDHEX on Gefitinib-resistant Human Non-small Cell Lung Cancer. J. Cancer.

[4] Lalle M., Camerini S., Cecchetti S., Finelli R., Sferra G., Müller J., Ricci G., Pozio E. (2015). The FAD-dependent glycerol-3-phosphate dehydrogenase of Giardia duodenalis: An unconventional enzyme that interacts with the g14-3-3 and it is a target of the antitumoral compound NBDHEX. Front. Microbiol..

[5] Camerini S., Bocedi A., Cecchetti S., Casella M., Carbo M., Morea V., Pozio E., Ricci G., Lalle M. (2017). Proteomic and functional analyses reveal pleiotropic action of the anti-tumoral compound NBDHEX in Giardia duodenalis. Int. J. Parasitol. Drugs Drug Resist..

[6] Brogi S., Fiorillo A., Chemi G., Butini S., Lalle M., Ilari A., Gemma S., Campiani G. (2017). Structural characterization of Giardia duodenalis thioredoxin reductase (gTrxR) and computational analysis of its interaction with NBDHEX. Eur. J. Med. Chem..

[7] Fulci C., Rotili D., De Luca A., Stella L., Morozzo Della Rocca B., Forgione M., Di Paolo V., Mai A., Falconi M., Quintieri L. (2017). A new nitrobenzoxadiazole-based GSTP1-1 inhibitor with a previously unheard of mechanism of action and high stability. J. Enzyme Inhib. Med. Chem..

[8] Di Paolo V., Fulci C., Rotili D., Sciarretta F., Lucidi A., Morozzo Della Rocca B., De Luca A., Rosato A., Quintieri L., Caccuri A.M. (2019). Synthesis and characterisation of a new benzamide-containing nitrobenzoxadiazole as a GSTP1-1 inhibitor endowed with high stability to metabolic hydrolysis. J. Enzyme Inhib. Med. Chem..

[9] WHO World Malaria Report 2021. https://www.who.int/teams/global-malaria-programme/reports/world-malaria-report-2021.

[10] Uwimana A., Umulisa N., Venkatesan M., Svigel S.S., Zhou Z., Munyaneza T., Habimana R.M., Rucogoza A., Moriarty L.F., Sandford R. (2021). Association of Plasmodium falciparum kelch13 R561H genotypes with delayed parasite clearance in Rwanda: An open-label, single-arm, multicentre, therapeutic efficacy study. Lancet Infect. Dis..

[11] Adjalley S.H., Johnston G.L., Li T., Eastman R.T., Ekland E.H., Eappen A.G., Richman A., Sim B.K., Lee M.C., Hoffman S.L. (2011). Quantitative assessment of Plasmodium falciparum sexual development reveals potent transmission-blocking activity by methylene blue. Proc. Natl. Acad. Sci. USA.

[12] Plouffe D.M., Wree M., Du A.Y., Meister S., Li F., Patra K., Lubar A., Okitsu S.L., Flannery E.L., Kato N. (2016). High-Throughput Assay and Discovery of Small Molecules that Interrupt Malaria Transmission. Cell Host Microbe.

[13] Siciliano G., Santha Kumar T.R., Bona R., Camarda G., Calabretta M.M., Cevenini L., Davioud-Charvet E., Becker K., Cara A., Fidock D.A. (2017). A high susceptibility to redox imbalance of the transmissible stages of Plasmodium falciparum revealed with a luciferase-based mature gametocyte assay. Mol. Microbiol..

[14] D’Alessandro S., Silvestrini F., Dechering K., Corbett Y., Parapini S., Timmerman M., Galastri L., Basilico N., Sauerwein R., Alano P. (2013). A Plasmodium falciparum screening assay for anti-gametocyte drugs based on parasite lactate dehydrogenase detection. J. Antimicrob. Chemother..

[15] Cevenini L., Camarda G., Michelini E., Siciliano G., Calabretta M.M., Bona R., Kumar T.R., Cara A., Branchini B.R., Fidock D.A. (2014). Multicolor bioluminescence boosts malaria research: Quantitative dual-color assay and single-cell imaging in Plasmodium falciparum parasites. Anal. Chem..

[16] Dechering K.J., Duerr H.P., Koolen K.M.J., Gemert G.V., Bousema T., Burrows J., Leroy D., Sauerwein R.W. (2017). Modelling mosquito infection at natural parasite densities identifies drugs targeting EF2, PI4K or ATP4 as key candidates for interrupting malaria transmission. Sci. Rep..

[17] Parkinson A., Ogilvie B.W., Buckley D.B., Kazmi F., Czerwinski M., Parkinson O. (2013). Biotransformation of xenobiotics. Casarett & Doull’s Toxicology: The Basic Science of Poisons.

[18] Robien M.A., Bosch J., Buckner F.S., Van Voorhis W.C., Worthey E.A., Myler P., Mehlin C., Boni E.E., Kalyuzhniy O., Anderson L. (2006). Crystal structure of glyceraldehyde-3-phosphate dehydrogenase from Plasmodium falciparum at 2.25 A resolution reveals intriguing extra electron density in the active site. Proteins.

[19] More K.R., Kaur I., Giai Gianetto Q., Invergo B.M., Chaze T., Jain R., Huon C., Gutenbrunner P., Weisser H., Matondo M. (2020). Phosphorylation-Dependent Assembly of a 14-3-3 Mediated Signaling Complex during Red Blood Cell Invasion by Plasmodium falciparum Merozoites. mBio.

[20] Lalle M., Currà C., Ciccarone F., Pace T., Cecchetti S., Fantozzi L., Ay B., Breton C.B., Ponzi M. (2011). Dematin, a component of the erythrocyte membrane skeleton, is internalized by the malaria parasite and associates with Plasmodium 14-3-3. J. Biol. Chem..

[21] Dastidar E.G., Dzeyk K., Krijgsveld J., Malmquist N.A., Doerig C., Scherf A., Lopez-Rubio J.J. (2013). Comprehensive histone phosphorylation analysis and identification of Pf14-3-3 protein as a histone H3 phosphorylation reader in malaria parasites. PLoS ONE.

[22] Lalle M., Fiorillo A. (2019). The protein 14-3-3: A functionally versatile molecule in Giardia duodenalis. Adv. Parasitol..

[23] Wu X., Rapoport T.A. (2018). Mechanistic insights into ER-associated protein degradation. Curr. Opin. Cell. Biol..

[24] Spork S., Hiss J.A., Mandel K., Sommer M., Kooij T.W., Chu T., Schneider G., Maier U.G., Przyborski J.M. (2009). An unusual ERAD-like complex is targeted to the apicoplast of Plasmodium falciparum. Eukaryot. Cell.

[25] Wong W., Bai X.C., Brown A., Fernandez I.S., Hanssen E., Condron M., Tan Y.H., Baum J., Scheres S.H. (2014). Cryo-EM structure of the Plasmodium falciparum 80S ribosome bound to the anti-protozoan drug emetine. Elife.

[26] Wong W., Bai X.C., Sleebs B.E., Triglia T., Brown A., Thompson J.K., Jackson K.E., Hanssen E., Marapana D.S., Fernandez I.S. (2017). Mefloquine targets the Plasmodium falciparum 80S ribosome to inhibit protein synthesis. Nat. Microbiol..

[27] Fidock D.A. (2010). Drug discovery: Priming the antimalarial pipeline. Nature.

[28] Lucantoni L., Silvestrini F., Signore M., Siciliano G., Eldering M., Dechering K.J., Avery V.M., Alano P. (2015). A simple and predictive phenotypic High Content Imaging assay for Plasmodium falciparum mature gametocytes to identify malaria transmission blocking compounds. Sci. Rep..

[29] Sun W., Tanaka T.Q., Magle C.T., Huang W., Southall N., Huang R., Dehdashti S.J., McKew J.C., Williamson K.C., Zheng W. (2014). Chemical signatures and new drug targets for gametocytocidal drug development. Sci. Rep..

[30] Wadi I., Nath M., Anvikar A.R., Singh P., Sinha A. (2019). Recent advances in transmission-blocking drugs for malaria elimination. Future Med. Chem..

[31] Wadi I., Singh P., Nath M., Anvikar A.R., Sinha A. (2020). Malaria transmission-blocking drugs: Implications and future perspectives. Future Med. Chem..

[32] Liebau E., De Maria F., Burmeister C., Perbandt M., Turella P., Antonini G., Federici G., Giansanti F., Stella L., Lo Bello M. (2005). Cooperativity and pseudo-cooperativity in the glutathione S-transferase from Plasmodium falciparum. J. Biol. Chem..

[33] Pierce A., Mirzaei H., Muller F., De Waal E., Taylor A.B., Leonard S., Van Remmen H., Regnier F., Richardson A., Chaudhuri A. (2008). GAPDH is conformationally and functionally altered in association with oxidative stress in mouse models of amyotrophic lateral sclerosis. J. Mol. Biol..

[34] Stevers L.M., Sijbesma E., Botta M., MacKintosh C., Obsil T., Landrieu I., Cau Y., Wilson A.J., Karawajczyk A., Eickhoff J. (2018). Modulators of 14-3-3 Protein-Protein Interactions. J. Med. Chem..

[35] Sehnke P.C., Laughner B., Cardasis H., Powell D., Ferl R.J. (2006). Exposed loop domains of complexed 14-3-3 proteins contribute to structural diversity and functional specificity. Plant Physiol..

[36] Goulielmaki E., Kaforou S., Venugopal K., Loukeris T.G., Siden-Kiamos I., Koussis K. (2018). Distinct effects of HIV protease inhibitors and ERAD inhibitors on zygote to ookinete transition of the malaria parasite. Mol. Biochem. Parasitol..

[37] Watts G.D., Wymer J., Kovach M.J., Mehta S.G., Mumm S., Darvish D., Pestronk A., Whyte M.P., Kimonis V.E. (2004). Inclusion body myopathy associated with Paget disease of bone and frontotemporal dementia is caused by mutant valosin-containing protein. Nat. Genet..

[38] Nogales E., Downing K.H., Amos L.A., Löwe J. (1998). Tubulin and FtsZ form a distinct family of GTPases. Nat. Struct. Biol..

[39] Schwank S., Sutherland C.J., Drakeley C.J. (2010). Promiscuous expression of α-tubulin II in maturing male and female Plasmodium falciparum gametocytes. PLoS ONE.

[40] Silvestrini F., Lasonder E., Olivieri A., Camarda G., van Schaijk B., Sanchez M., Younis Younis S., Sauerwein R., Alano P. (2010). Protein export marks the early phase of gametocytogenesis of the human malaria parasite Plasmodium falciparum. Mol. Cell. Proteom..

[41] Jida M., Sanchez C.P., Urgin K., Ehrhardt K., Mounien S., Geyer A., Elhabiri M., Lanzer M., Davioud-Charvet E. (2017). A Redox-Active Fluorescent pH Indicator for Detecting Plasmodium falciparum Strains with Reduced Responsiveness to Quinoline Antimalarial Drugs. ACS Infect. Dis..

[42] De Luca A., Rotili D., Carpanese D., Lenoci A., Calderan L., Scimeca M., Mai A., Bonanno E., Rosato A., Geroni C. (2015). A novel orally active water-soluble inhibitor of human glutathione transferase exerts a potent and selective antitumor activity against human melanoma xenografts. Oncotarget.

[43] Di Paolo V., Fulci C., Rotili D., De Luca A., Tomassi S., Serra M., Scimeca M., Geroni C., Quintieri L., Caccuri A.M. (2020). Characterization of water-soluble esters of nitrobenzoxadiazole-based GSTP1-1 inhibitors for cancer treatment. Biochem. Pharmacol..

[44] Ng C.L., Siciliano G., Lee M.C., de Almeida M.J., Corey V.C., Bopp S.E., Bertuccini L., Wittlin S., Kasdin R.G., Le Bihan A. (2016). CRISPR-Cas9-modified pfmdr1 protects Plasmodium falciparum asexual blood stages and gametocytes against a class of piperazine-containing compounds but potentiates artemisinin-based combination therapy partner drugs. Mol. Microbiol..

[45] Rotili D., De Luca A., Tarantino D., Pezzola S., Forgione M., Morozzo Della Rocca B., Falconi M., Mai A., Caccuri A.M. (2015). Synthesis and structure--activity relationship of new cytotoxic agents targeting human glutathione-S-transferases. Eur. J. Med. Chem..

[46] Lo Bello M., Battistoni A., Mazzetti A.P., Board P.G., Muramatsu M., Federici G., Ricci G. (1995). Site-directed mutagenesis of human glutathione transferase P1-1. Spectral, kinetic, and structural properties of Cys-47 and Lys-54 mutants. J. Biol. Chem..

[47] Harwaldt P., Rahlfs S., Becker K. (2002). Glutathione S-transferase of the malarial parasite Plasmodium falciparum: Characterization of a potential drug target. Biol. Chem..

[48] Habig W.H., Jakoby W.B. (1981). Assays for differentiation of glutathione S-transferases. Methods Enzymol..

[49] Walliker D., Quakyi I.A., Wellems T.E., McCutchan T.F., Szarfman A., London W.T., Corcoran L.M., Burkot T.R., Carter R. (1987). Genetic analysis of the human malaria parasite Plasmodium falciparum. Science.

[50] Vos M.W., Stone W.J., Koolen K.M., van Gemert G.J., van Schaijk B., Leroy D., Sauerwein R.W., Bousema T., Dechering K.J. (2015). A semi-automated luminescence based standard membrane feeding assay identifies novel small molecules that inhibit transmission of malaria parasites by mosquitoes. Sci. Rep..

[51] Trager W., Jensen J.B. (1976). Human malaria parasites in continuous culture. Science.

[52] Gupta S.K., Schulman S., Vanderberg J.P. (1985). Stage-dependent toxicity of N-acetyl-glucosamine to Plasmodium falciparum. J. Protozool..

[53] Shevchenko A., Wilm M., Vorm O., Mann M. (1996). Mass spectrometric sequencing of proteins silver-stained polyacrylamide gels. Anal. Chem..

[54] Perez-Riverol Y., Csordas A., Bai J., Bernal-Llinares M., Hewapathirana S., Kundu D.J., Inuganti A., Griss J., Mayer G., Eisenacher M. (2019). The PRIDE database and related tools and resources in 2019: Improving support for quantification data. Nucleic Acids Res..

[55] Pettersen E.F., Goddard T.D., Huang C.C., Couch G.S., Greenblatt D.M., Meng E.C., Ferrin T.E. (2004). UCSF Chimera--a visualization system for exploratory research and analysis. J. Comput. Chem..

[56] Pettersen E.F., Goddard T.D., Huang C.C., Meng E.C., Couch G.S., Croll T.I., Morris J.H., Ferrin T.E. (2021). UCSF ChimeraX: Structure visualization for researchers, educators, and developers. Protein Sci..

