# Supplementary material for: The Nitrobenzoxadiazole Derivative NBDHEX Behaves as Plasmodium falciparum Gametocyte Selective Inhibitor with Malaria Parasite Transmission Blocking Activity
Source: Pharmaceuticals (Basel). 2022 Jan 29;15(2):168. doi: 10.3390/ph15020168 (PMC8875241; doi:10.3390/ph15020168)
Supplement: Supplementary file 1 [file pharmaceuticals-15-00168-s001.zip › pharmaceuticals-1555686-supplementary/pharmaceuticals-1555686 1H-NMR_13C-NMR_ESI-MS spectra compounds 1-4.pdf]

Compd 1 (CDCl3)

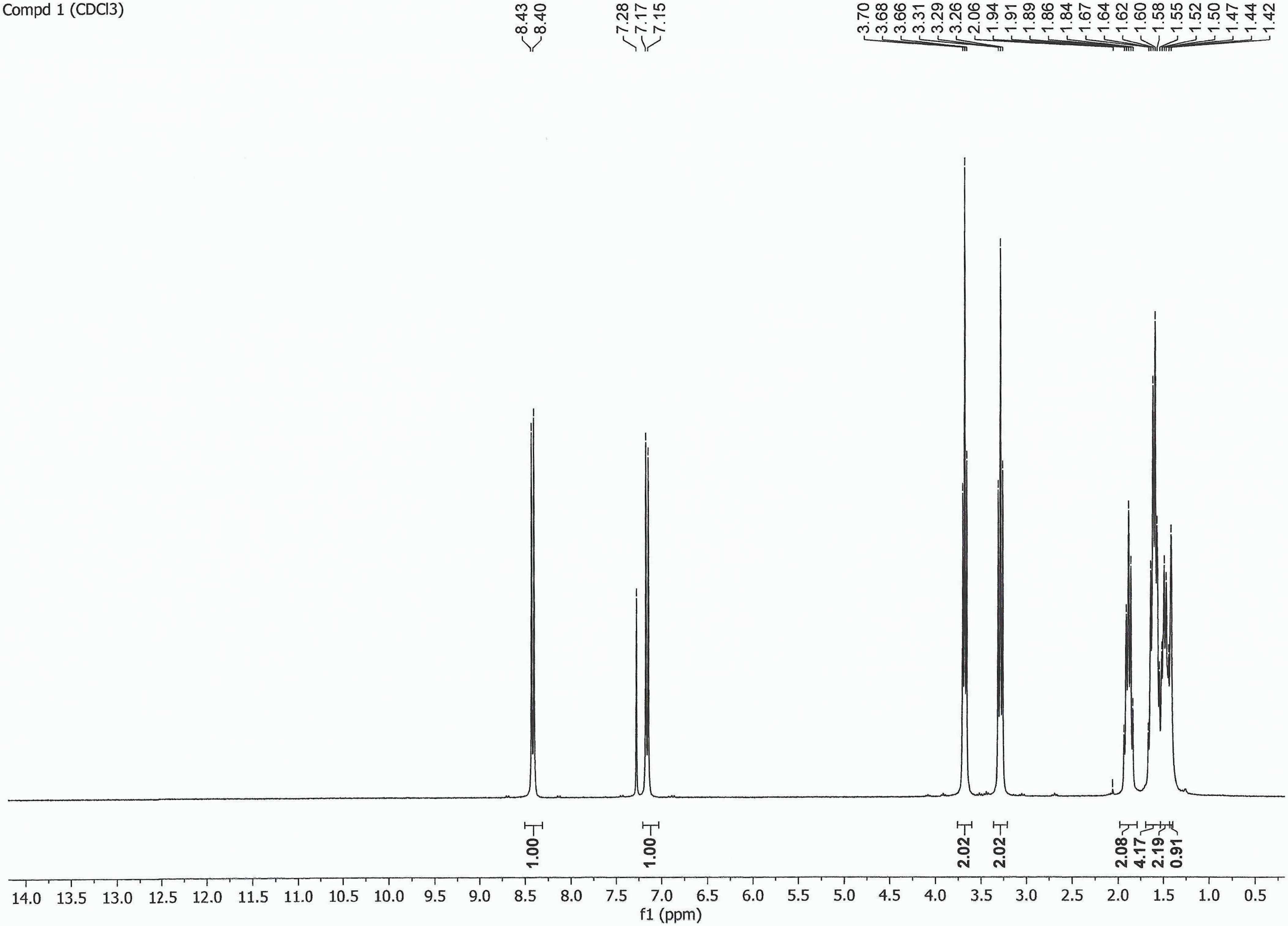

Compd 1 (CDCl3)

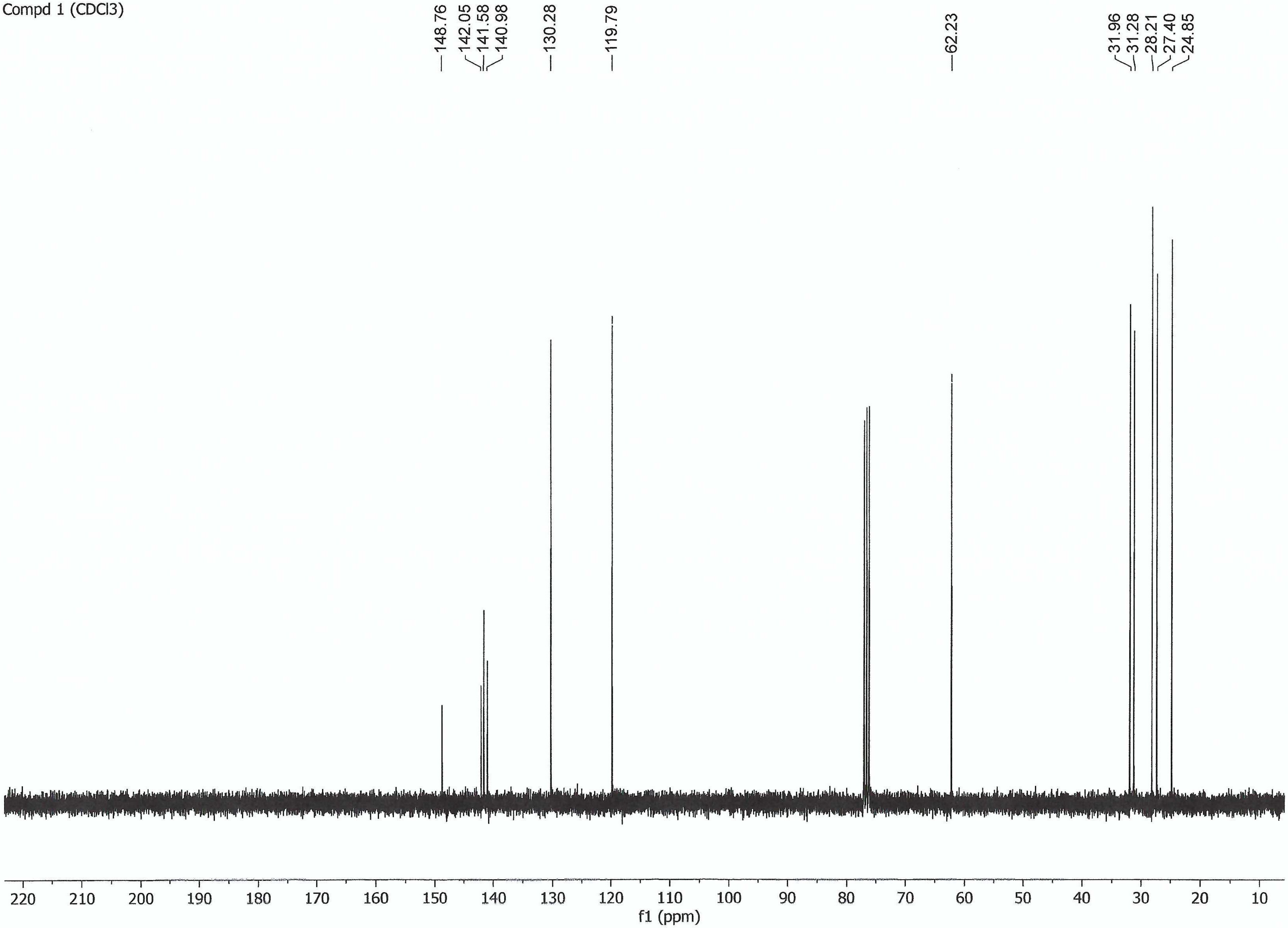

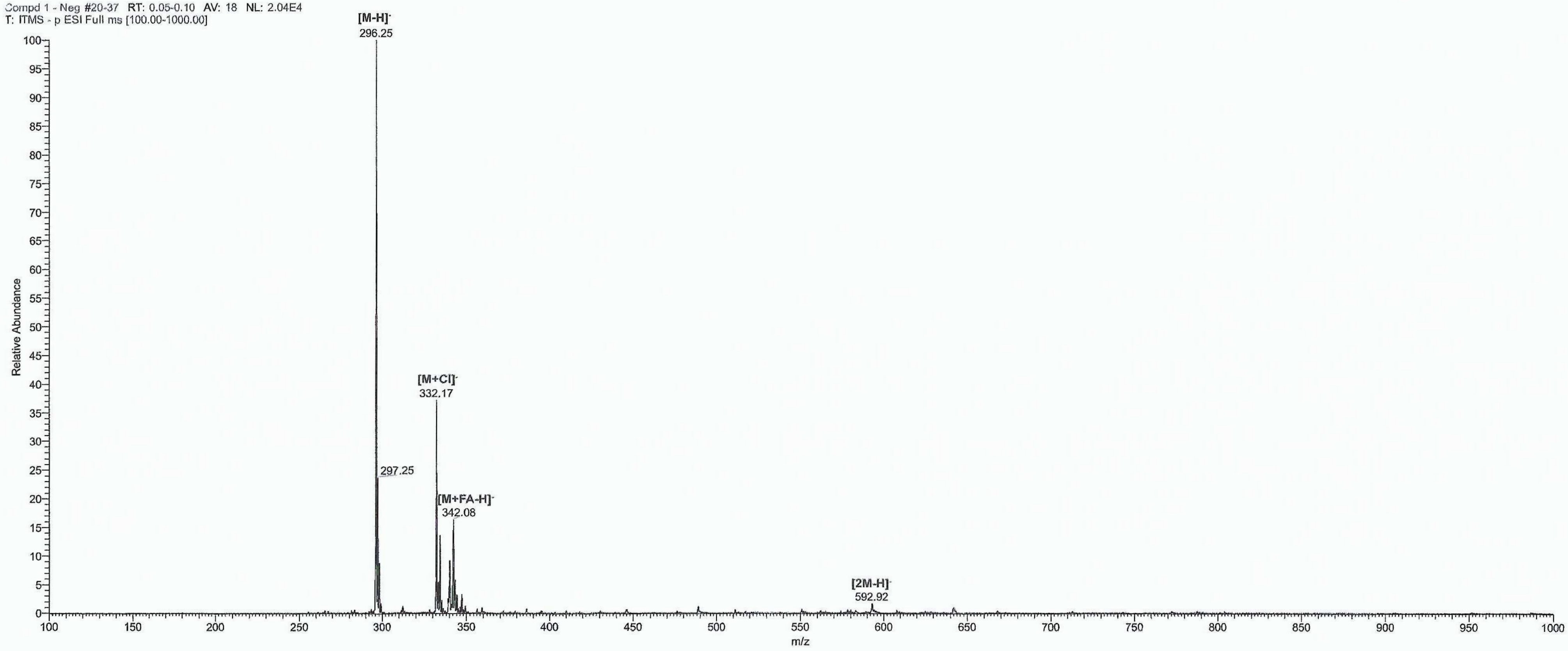

Compd 1 - Neg #20-37 RT: 0.05-0.10 AV: 18 NL: 2.04E4
T: ITMS - p ESI Full ms [100.00-1000.00]
[M-H]-
296.25
297.25
[M+Cl]-
332.17
[M+FA-H]-
342.08
[2M-H]-
592.92
Relative Abundance
m/z

Compd 2 (CD3OD)

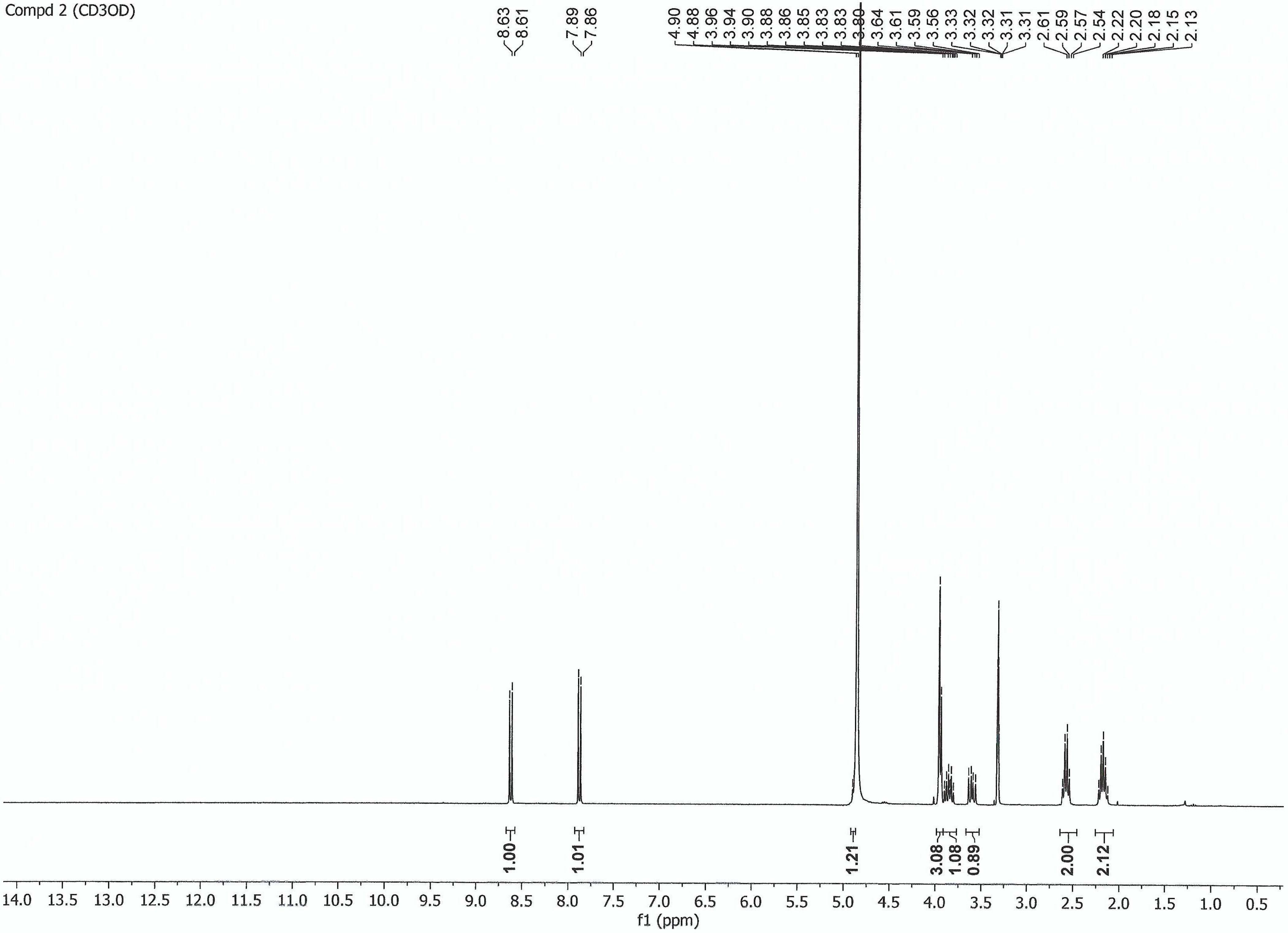

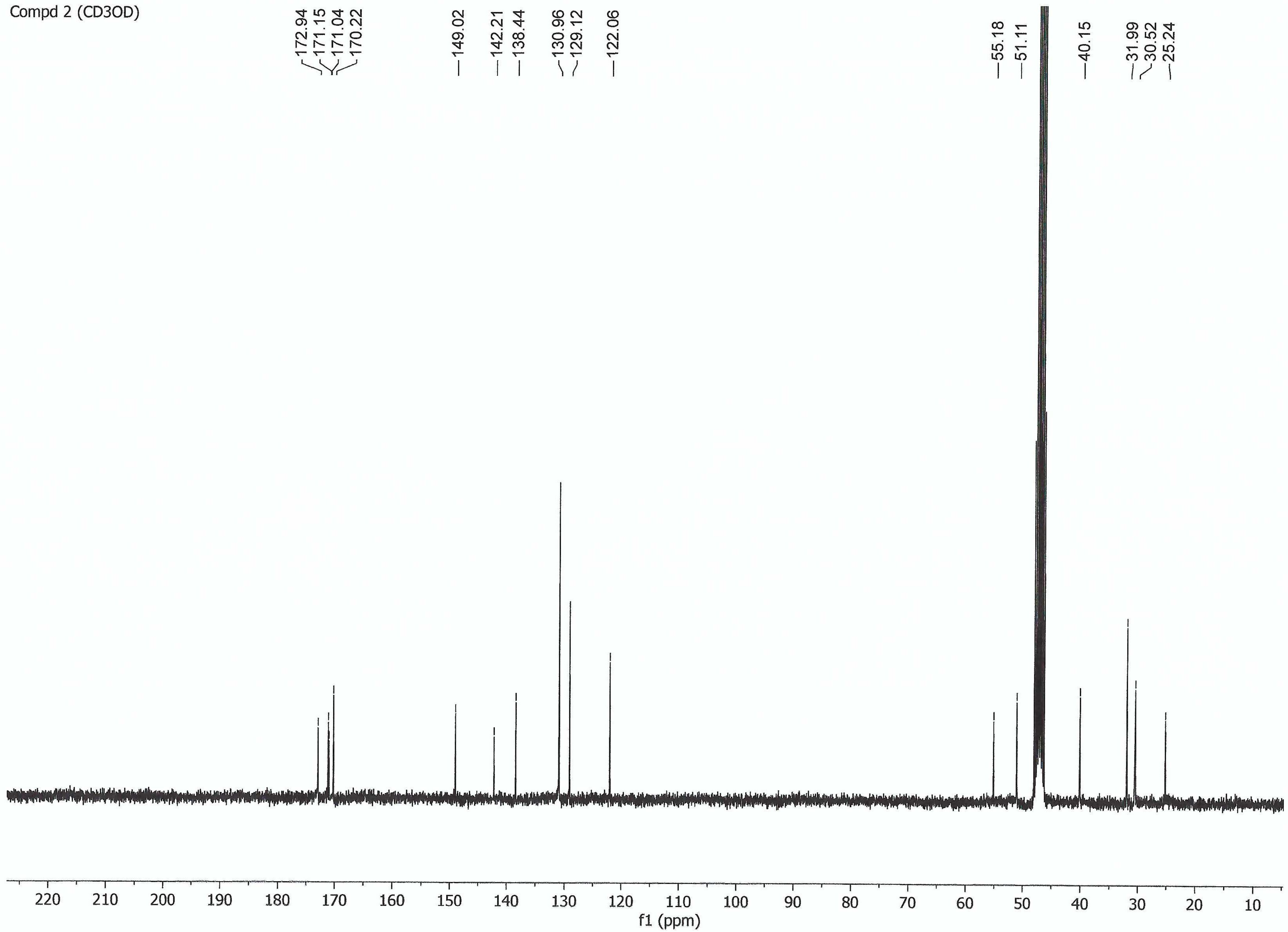
Compd 2 (CD3OD)
172.94
171.15
171.04
170.22
149.02
142.21
138.44
130.96
129.12
122.06
55.18
51.11
40.15
31.99
30.52
25.24
220
210
200
190
180
170
160
150
140
130
120
110
100
90
80
70
60
50
40
30
20
10
f1 (ppm)

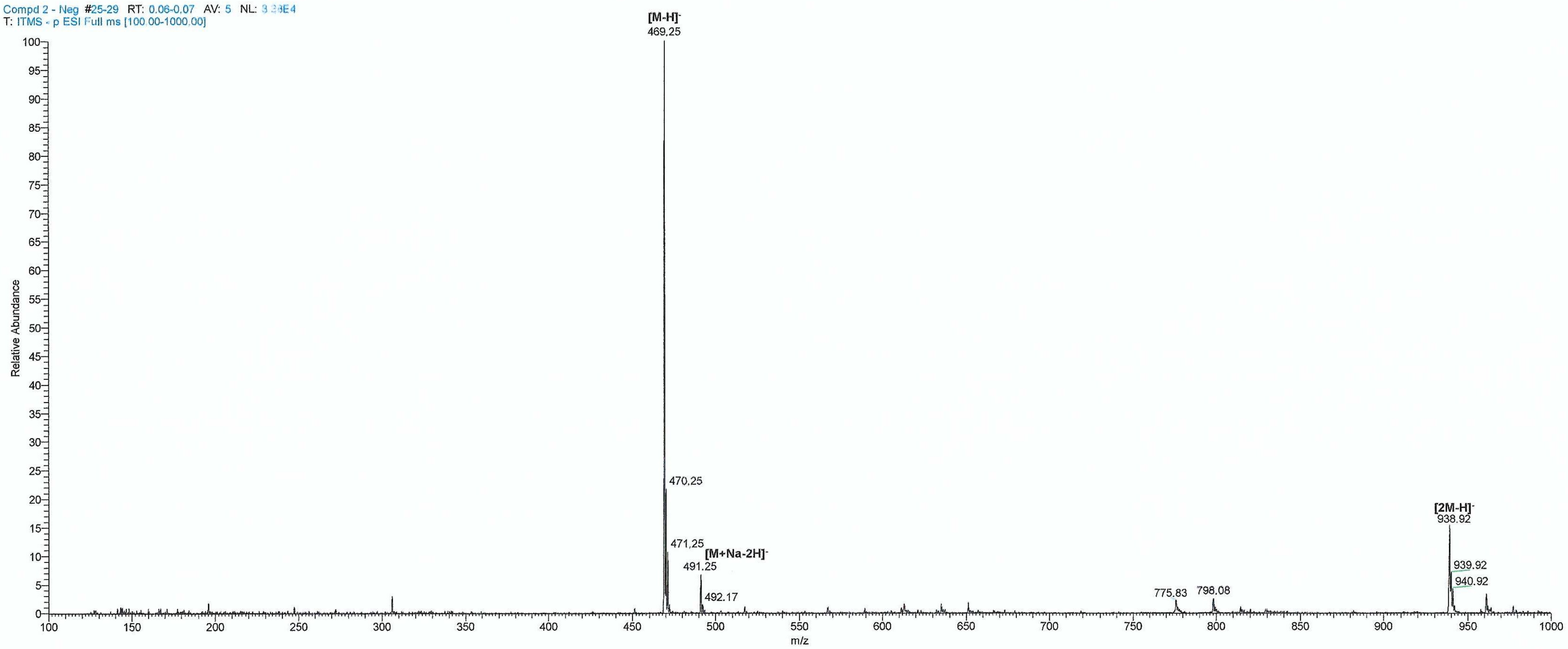
Compd 2 - Neg #25-29 RT: 0.06-0.07 AV: 5 NL:
T: ITMS - p ESI Full ms [100.00-1000.00]
Relative Abundance
m/z
[M-H]-
469.25
470.25
471.25
[M+Na-2H]-
491.25
492.17
775.83
798.08
[2M-H]-
938.92
939.92
940.92

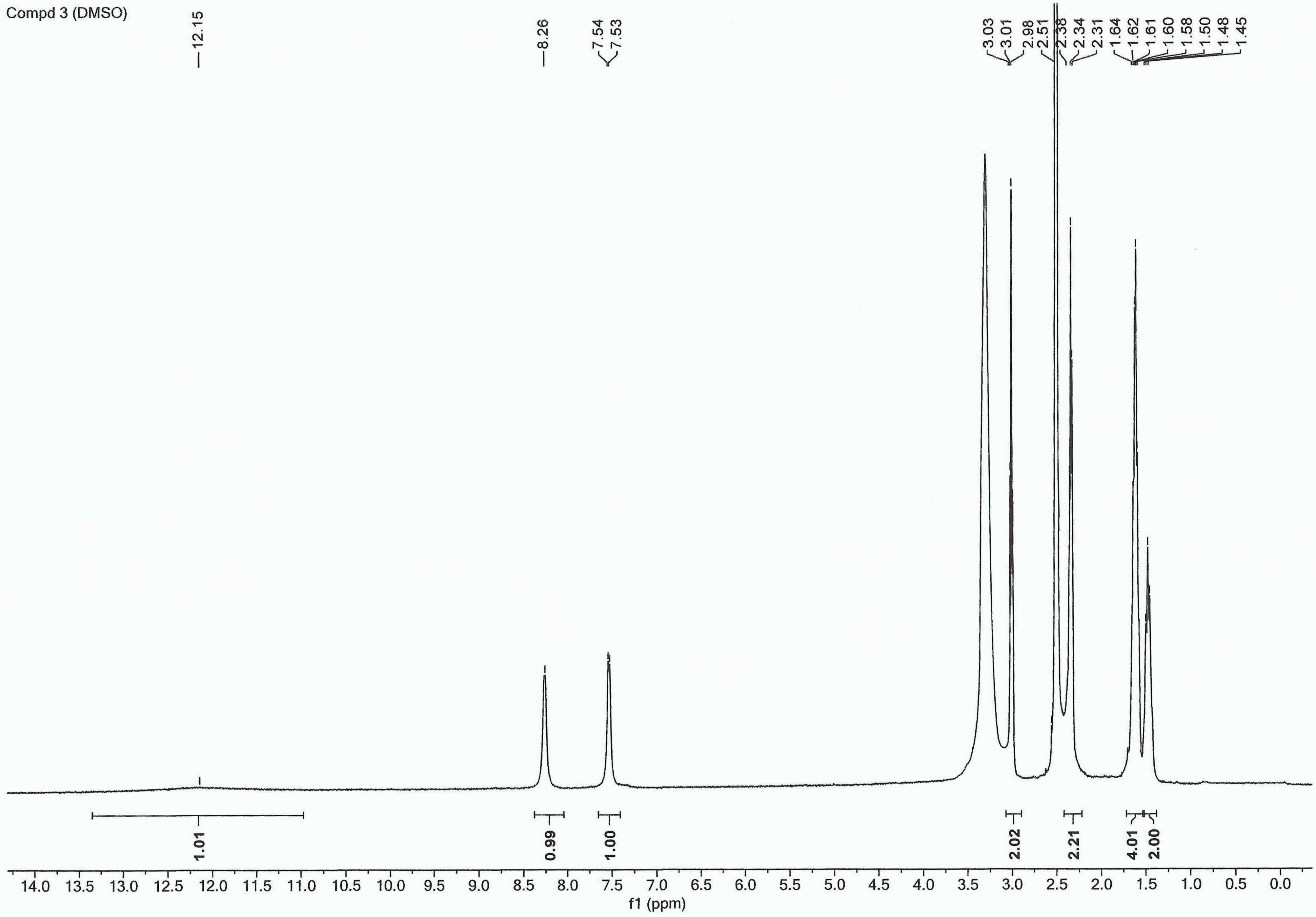
Compd 3 (DMSO)
12.15
8.26
7.54
7.53
3.03
3.01
2.98
2.51
2.38
2.34
2.31
1.64
1.62
1.61
1.60
1.58
1.50
1.48
1.45
1.01
0.99
1.00
2.02
2.21
4.01
2.00
14.0 13.5 13.0 12.5 12.0 11.5 11.0 10.5 10.0 9.5 9.0 8.5 8.0 7.5 7.0 6.5 6.0 5.5 5.0 4.5 4.0 3.5 3.0 2.5 2.0 1.5 1.0 0.5 0.0
f1 (ppm)

Compd 3 (DMSO)

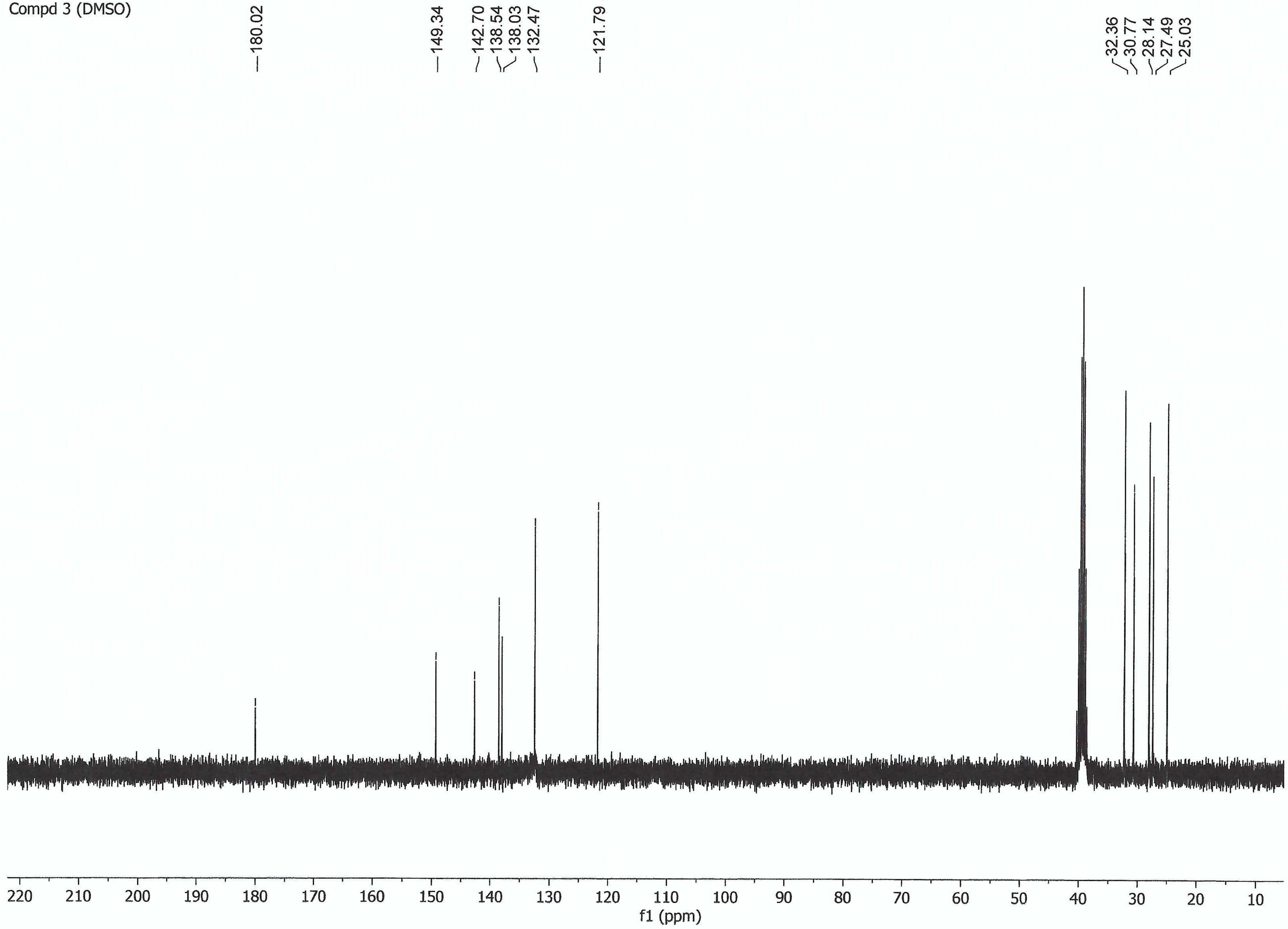
180.02
149.34
142.70
138.54
138.03
132.47
121.79
32.36
30.77
28.14
27.49
25.03
220 210 200 190 180 170 160 150 140 130 120 110 100 90 80 70 60 50 40 30 20 10
f1 (ppm)

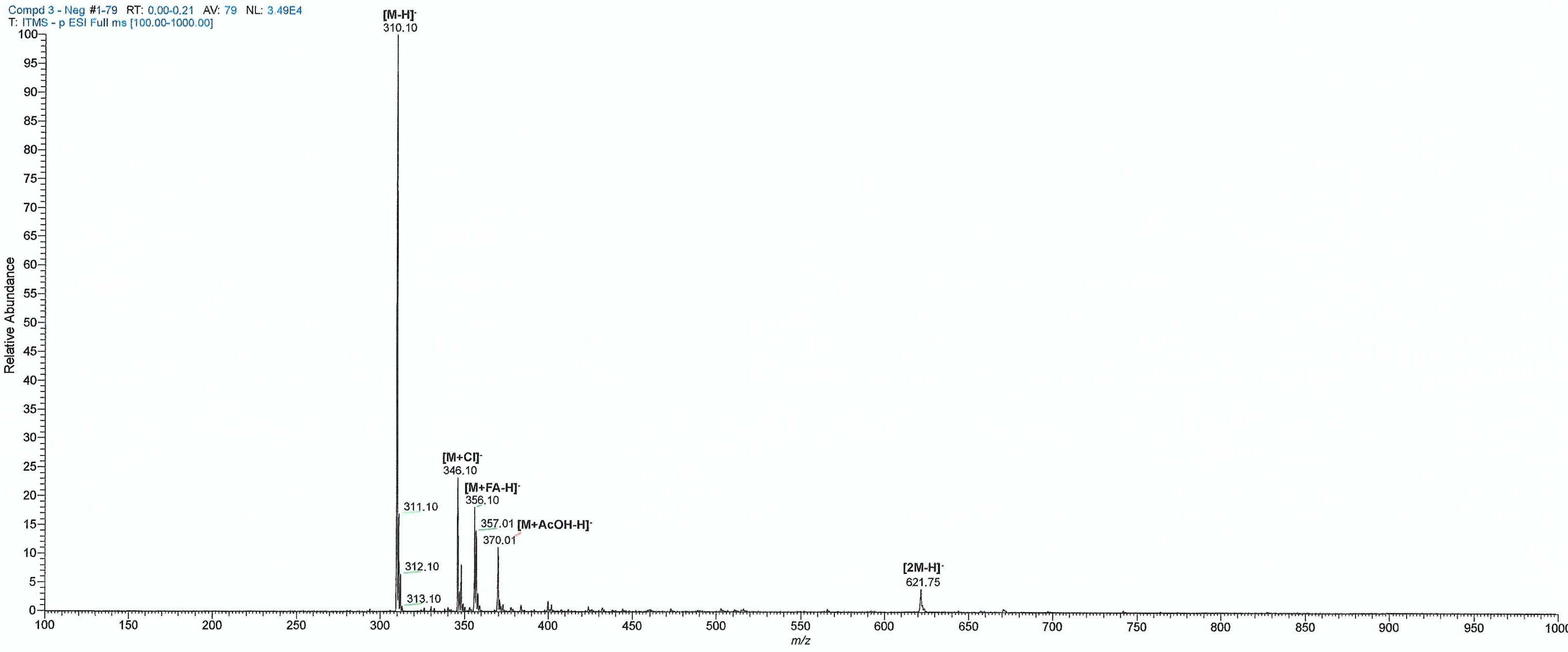
Compd 3 - Neg #1-79 RT: 0.00-0.21 AV: 79 NL: 3.49E4
T: ITMS - p ESI Full ms [100.00-1000.00]
[M-H]-
310.10
311.10
312.10
313.10
[M+Cl]-
346.10
[M+FA-H]-
356.10
357.01
[M+AcOH-H]-
370.01
[2M-H]-
621.75
Relative Abundance
m/z

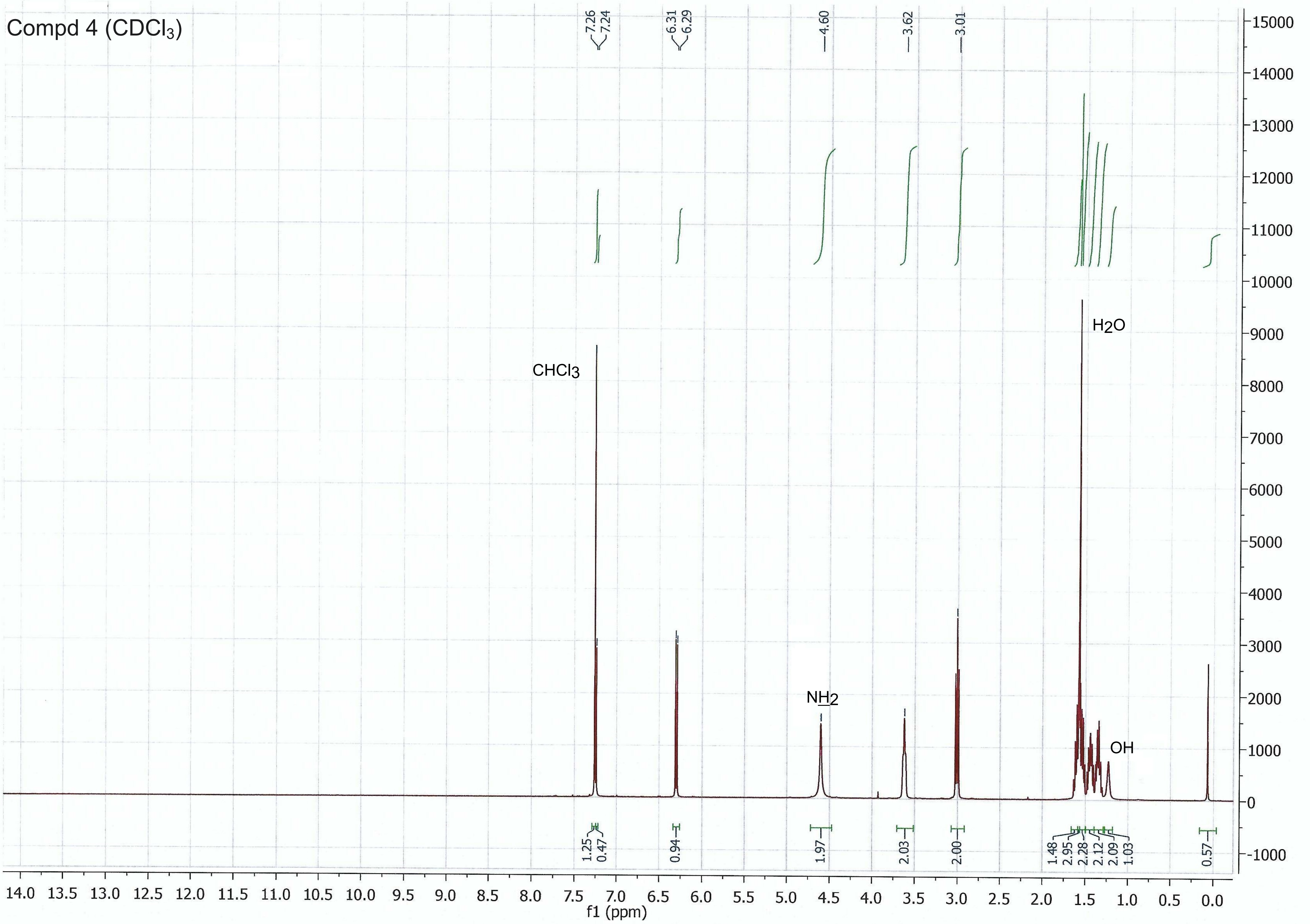

Compd 4 (CDCl3)
7.26
7.24
6.31
6.29
4.60
3.62
3.01
CHCl3
H2O
NH2
OH
1.25
0.47
0.94
1.97
2.03
2.00
1.48
2.95
2.28
2.12
2.09
1.03
0.57
f1 (ppm)

Compd 4 (CDCl3)

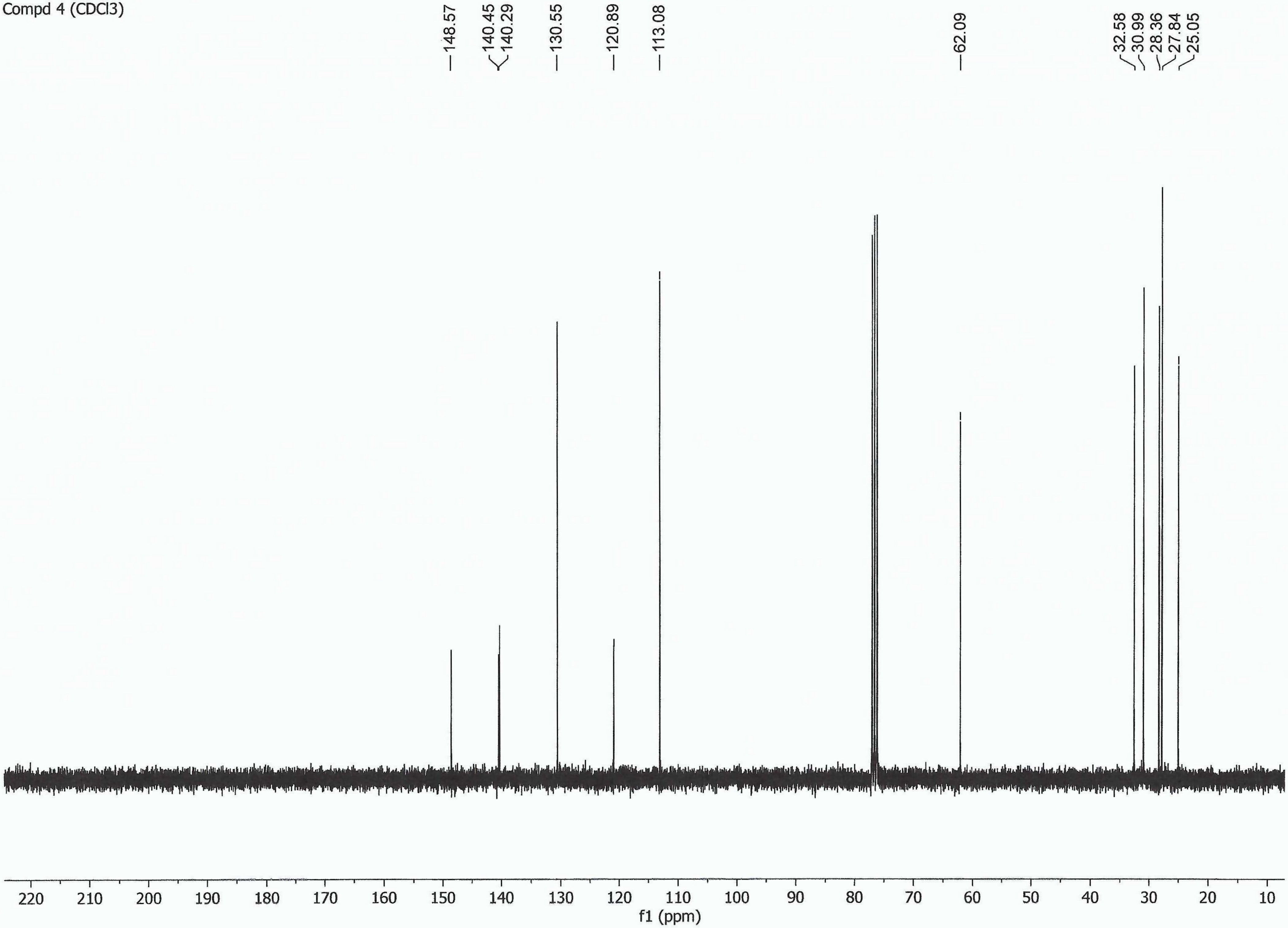

Dante_PR2A_acForm_171006132320 10/6/2017 1:23:20 PM

Compd 4

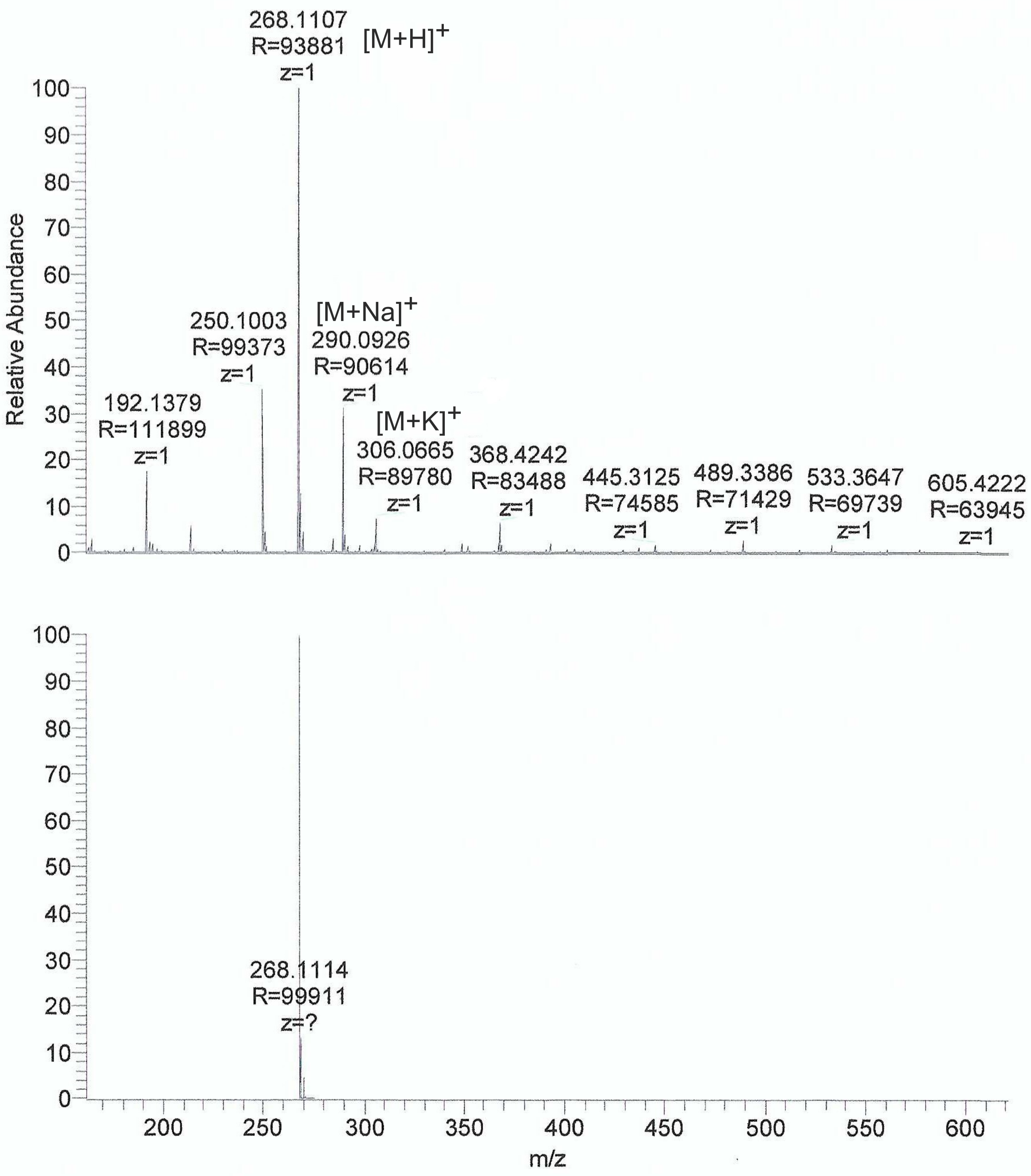

NL:
1.28E6
Dante_PR2A_acForm_171006
132320#2-42 RT: 0.04-0.64
AV: 41 T: FTMS + p ESI Full
ms [150.00-2000.00]

NL:
1.92E4
$C_{12}H_{17}N_3O_2S$ +H:
$C_{12}H_{18}N_3O_2S_1$
p (gss, s /p:40) Chrg 1
R: 100000 Res .Pwr . @FWHM
