# Supplementary material for: The Nitrobenzoxadiazole Derivative NBDHEX Behaves as Plasmodium falciparum Gametocyte Selective Inhibitor with Malaria Parasite Transmission Blocking Activity
Source: Pharmaceuticals (Basel). 2022 Jan 29;15(2):168. doi: 10.3390/ph15020168 (PMC8875241; doi:10.3390/ph15020168)
Supplement: Supplementary file 1 [file pharmaceuticals-15-00168-s001.zip › pharmaceuticals-1555686-supplementary/pharmaceuticals-1555686 Supplementary Figures S1-S9.pdf]

Supplementary Figures to:

The nitrobenzoxadiazole derivative NBDHEX behaves as *Plasmodium falciparum* gametocyte selective inhibitor with malaria parasite transmission blocking activity

Giulia Siciliano[a], Veronica Di Paolo[b], Dante Rotili[c], Rossella Migale[a], Francesca Pedini[d], Marialuisa Casella[e], Serena Camerini[e], Daniele Dalzoppo[b], Rob Henderson[f], Tonnie Huijs[f], Koen Dechering[f], Antonello Mai[c], Anna Maria Caccuri[g], Marco Lalle[a], Luigi Quintieri[b] and Pietro Alano[a]

a) Department of Infectious Diseases, Istituto Superiore di Sanità, 00161 Rome, Italy.
b) Department of Pharmaceutical and Pharmacological Sciences, University of Padua, Padua, Italy
c) Department of Chemistry and Technology of Drugs, "Sapienza" University of Rome, 00185 Rome, Italy
d) Department of Oncology and Molecular Medicine, Istituto Superiore di Sanità, 00161 Rome, Italy
e) Technical-Scientific Service, Core Facilities, Istituto Superiore di Sanità, 00161 Rome, Italy
f) TropIQ Health Sciences, 6534 AT Nijmegen, the Netherlands
g) Department of Chemical Sciences and Technologies, University of Tor Vergata, Rome, Italy

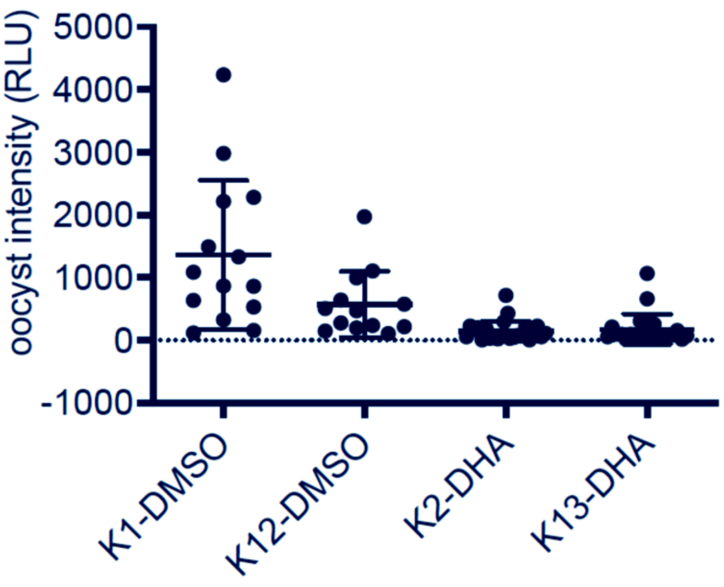

Figure S1. Positive (10μM Dihydroartemisinin) and negative (DMSO) controls in SMFA experiment.

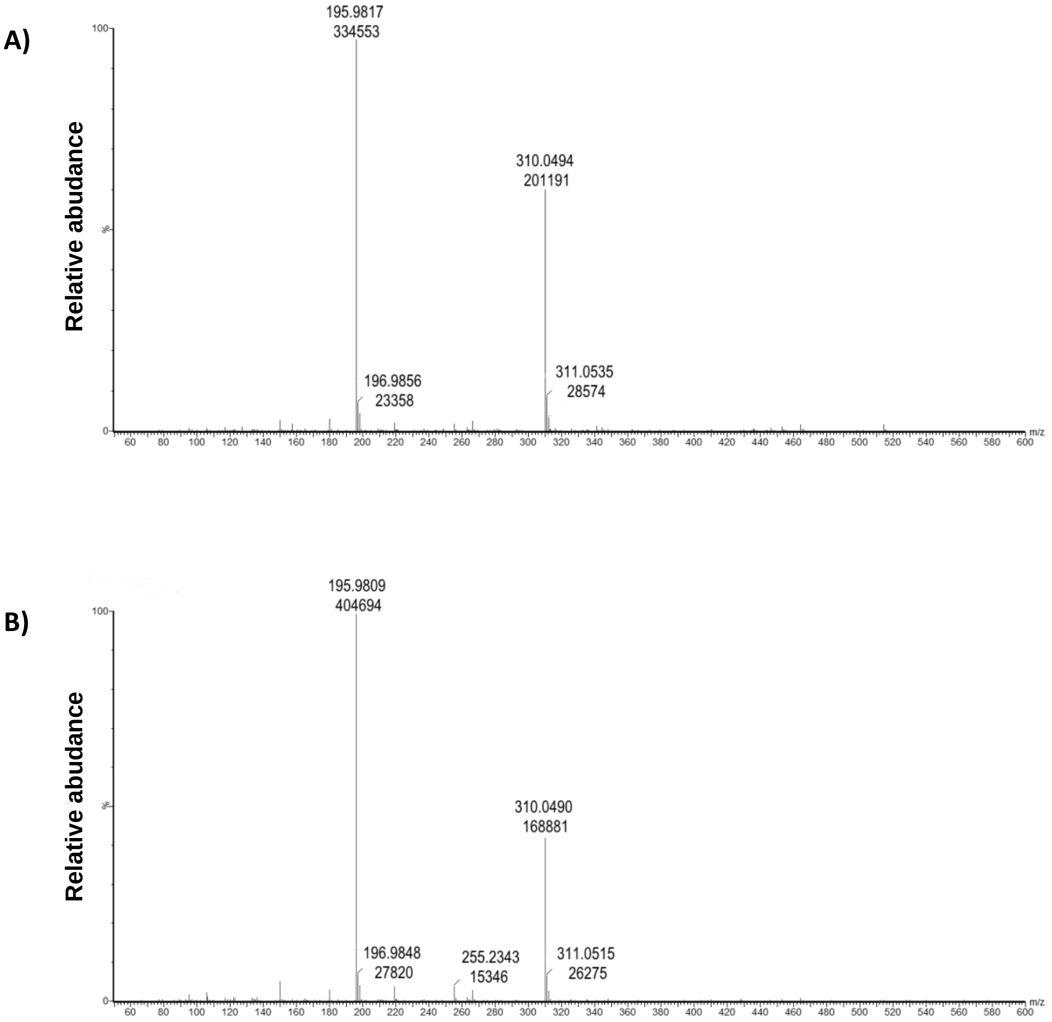

Figure S2. ESI-TOF-MS spectrum of peak "M" in the chromatogram of Fig. 3A and 3B. Mass spectra peaks at m/z 195.98 represent a fragment ion arising from the loss of C6H10O2 (carboxyhexyl moiety) from the parent ion (m/z = 310.04).

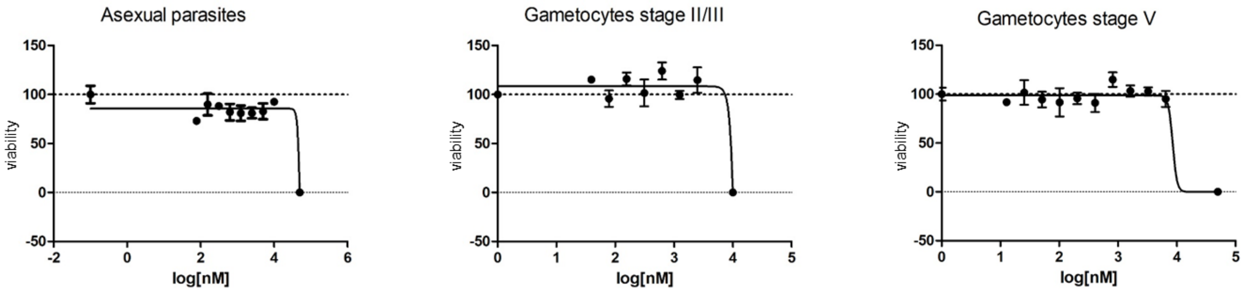

Figure S3. Representative concentration-response curves of the activity of **2** on asexual parasites, stage II/III and stage V gametocytes.

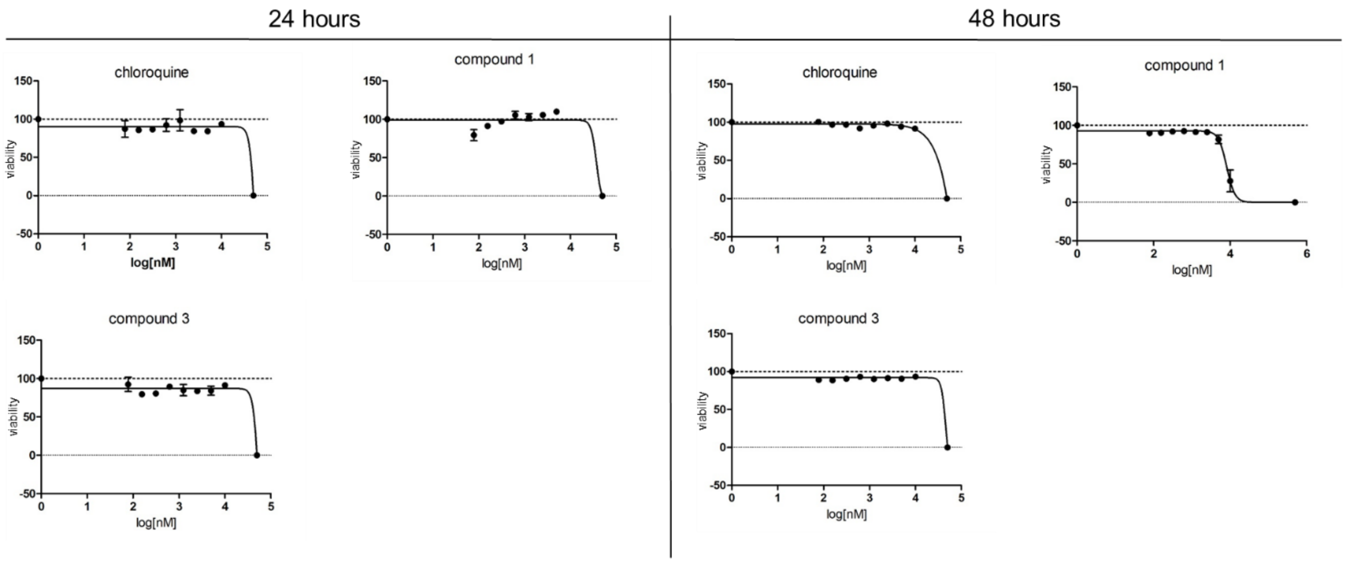

Figure S4. Representative concentration-response curves of the cytotoxic activity of chloroquine, of **1** and of **3** on VERO cells.

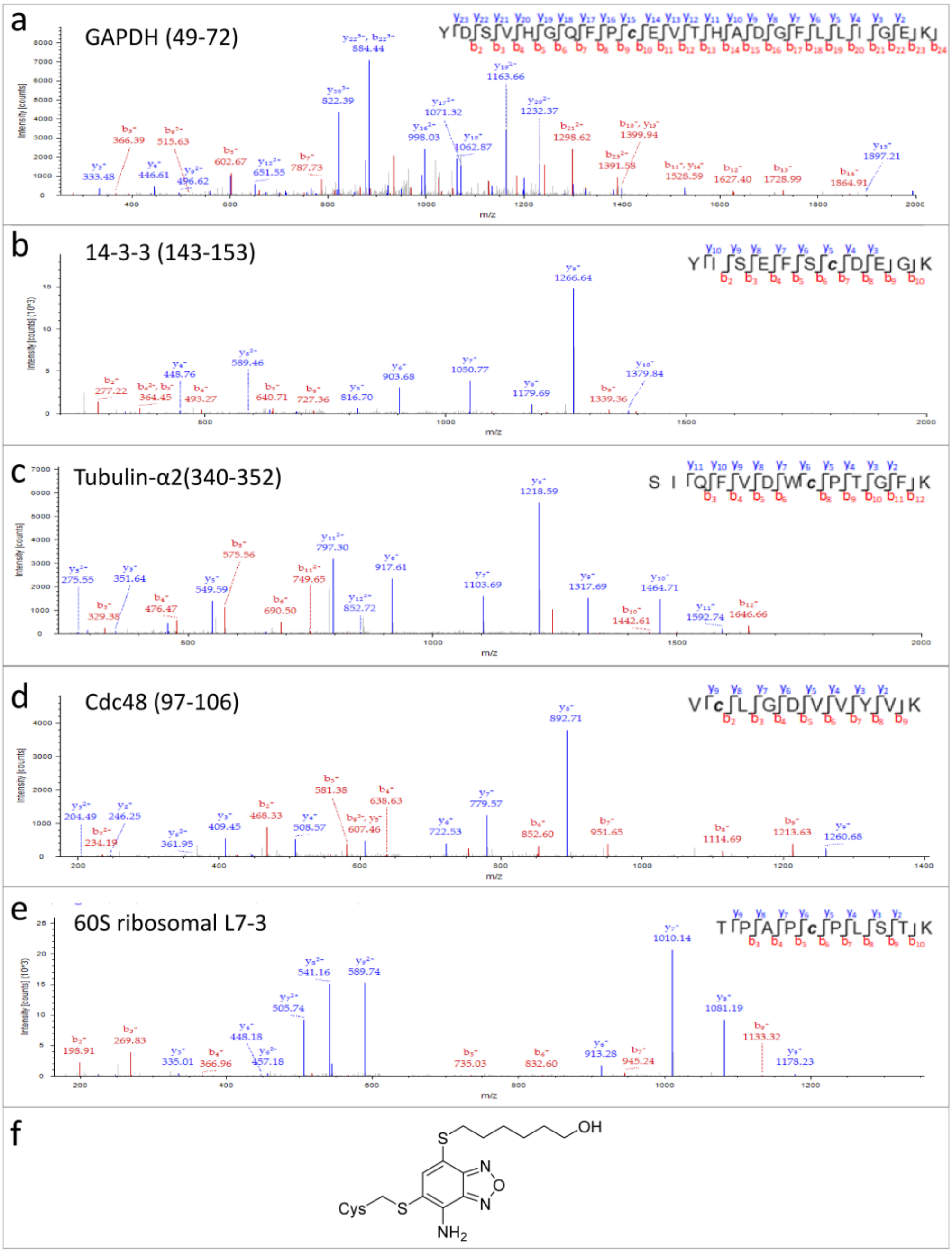

Figure S5. MS/MS spectra (panel a-e) of the modified peptides deriving from *P. falciparum* proteins GAPDH, 14-3-3I, Tubulin-α2, Cdc48, and 60S ribosomal L7-3, detected in the samples treated with **1**. In each panel the sequence of the peptide is reported; **c** (in bold) indicates the modified cysteine carrying a mass increase of 265Da corresponding to the adduct with7-nitro form of **1** (the formula is reported in panel f). The b and y series fragment ions reported on each sequence are detected in each spectrum. The red and blue peaks in each spectrum correspond to the b and y series fragment ions, respectively.

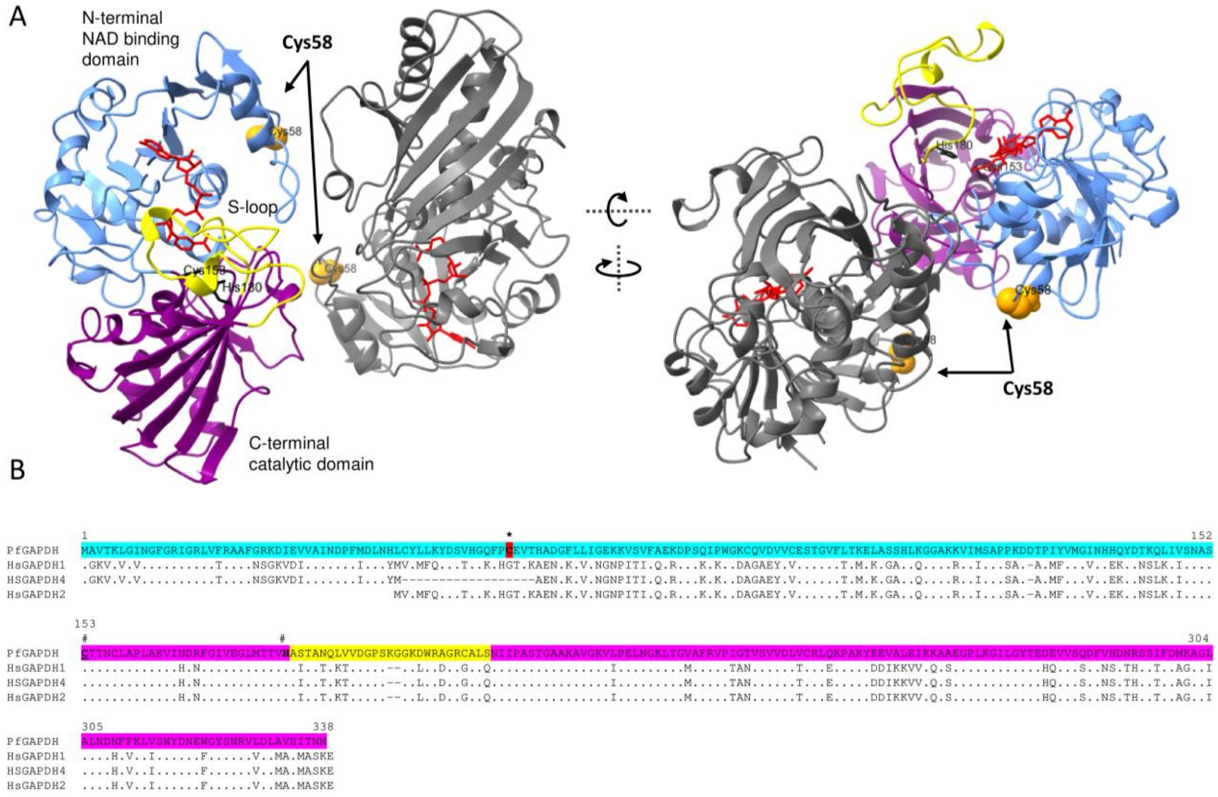

Figure S6. Localization of **1**-modified Cys58 in the PfGAPDH structure. A) Two protomers of the tetrameric PfGAPDH structure (PDB: 2B4T) are shown as cartoon. Different domains are coloured in one of the protomer: N-terminal NAD binding domain (light blue); S-loop (yellow); C-terminal catalytic domain (magenta). NAD molecules are shown (red). Side chains of the two catalytic residues Cys 153 and His180 are shown by ball and stick representation (black). Side chain of Cys58 is shown by spheres (orange). Figure was created with Chimera 1.15 [55]. B) Multiple sequence alignment of *Plasmodium falciparum* PfGAPDH (Acc. N. Q8IKK7) with *Homo sapiens* GAPDH isoform 1 (HsGAPDH1, NP_002037.2), isoform 2 (HsGAPDH2, NP_001243728.1) and isoform 4 (HsGAPDH4, NP_001344872.1). Sequence are numbered according to PfGAPDH. Different domains are coloured: N-terminal NAD binding domain (light blue); S-loop (yellow); C-terminal catalytic domain (magenta). NAD molecules are shown (red). Catalytic residues Cys 153 and His180 of PfGAPDH are underlined and marked by hashtags. Cys58 is highlighted (red) and marked by asterisk. Gaps are indicated by dashes. Conserved residues in the alignment are indicated by dots.

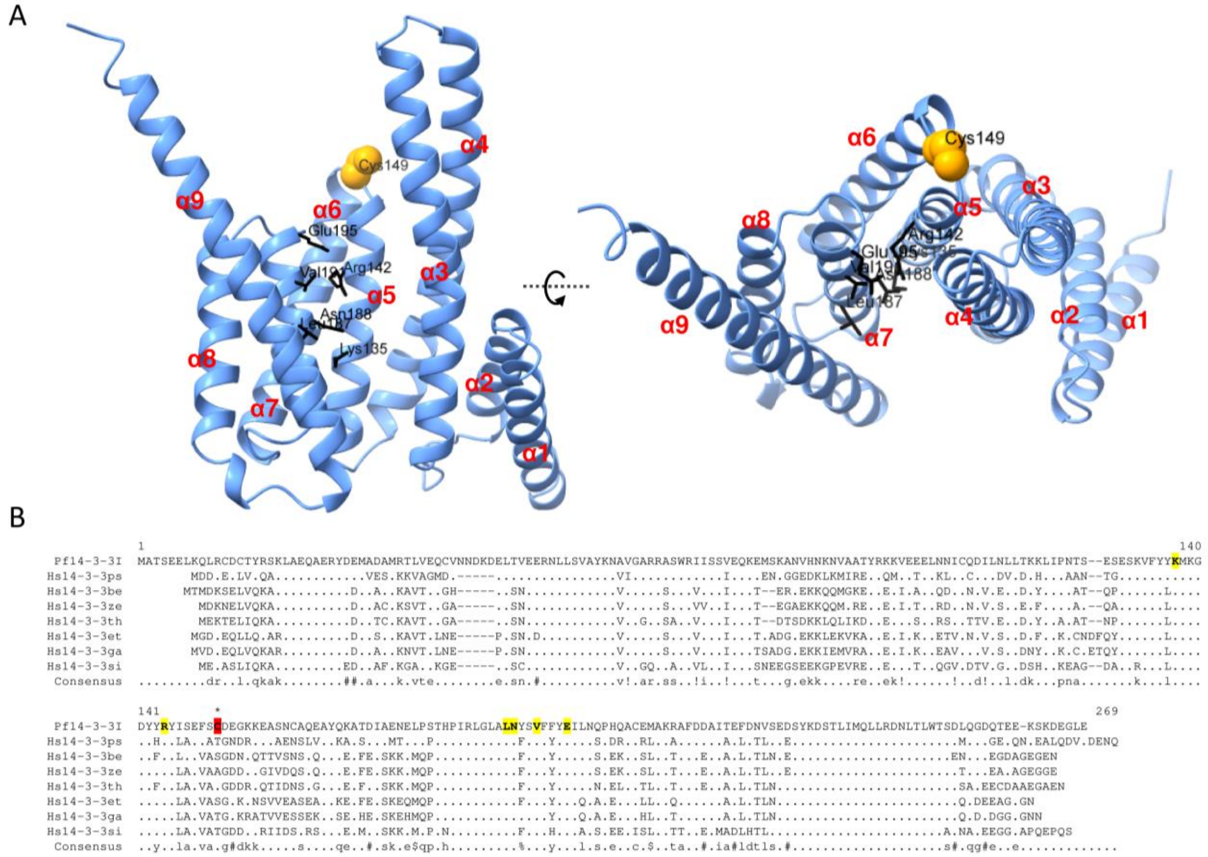

Figure S7. Localization of **1**-modified Cys149 in the Pf14-3-3I modelled structure. A) Homology model of Pf14-3-3I (light blue) (model AF-C0H4V6-F1) downloaded from AlphaFold database (https://alphafold.ebi.ac.uk/) is shown as cartoon. Alpha helices are marked (α1- α7). The side chains of the highly conserved residues (Lys135, Arg142, Leu187, Asn188, Val191 and Arg142) involved in phosphorylated target binding are shown by ball and stick representation (black). Side chain of Cys148 is shown by spheres (orange). Figure was created with ChimeraX [56]. B) Multiple sequence alignment of *Plasmodium falciparum* Pf14-3-3I (Acc. N. C0H4V6.1) with *Homo sapiens* 14-3-3 isoform beta (P31946.3, Hs14-3-3β), epsilon (P62258.1, Hs14-3-3ε), eta (Q04917.4, Hs14-3-3η), gamma (NP_036611.2; Hs14-3-3γ), sigma (P31947.1, Hs14-3-3σ) and theta (P27348.1, Hs14-3-3θ). Sequence are numbered according to Pf14-3-3I. Highly conserved residues (Lys135, Arg142, Leu187, Asn188, Val191 and Arg142) involved in phosphorylated target binding are in bold and highlighted (yellow). Cys149 is highlighted (red) and marked by asterisk. Gaps are indicated by dashes. Conserved residues in the alignment are indicated by dots.

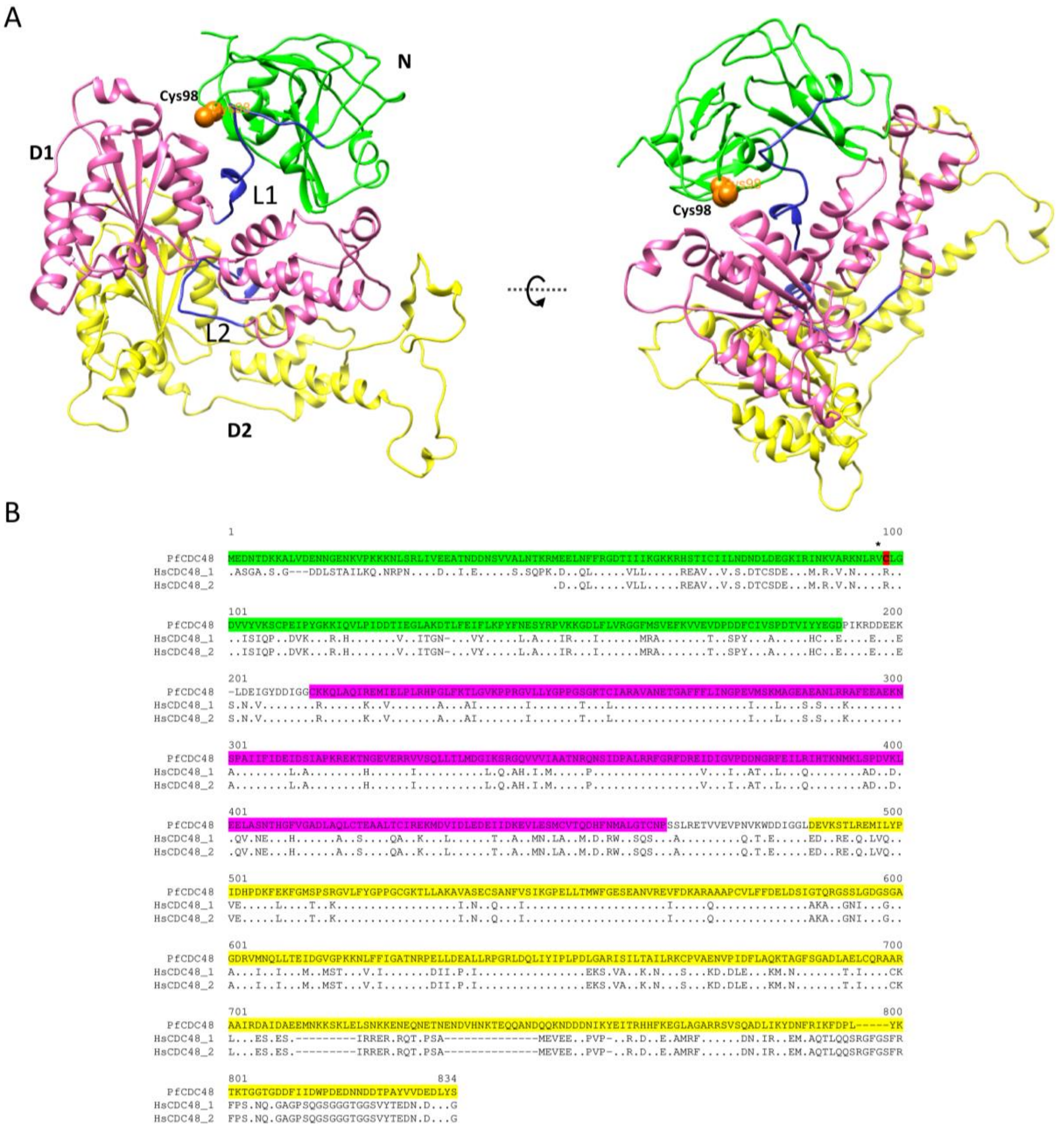

Figure S8. Localization of 1-modified Cys98 in the PfCdc48 modelled structure. A) Homology model of PfCdc48 was obtained using SwissModel (see Materials and Methods). A single protomer of the hexameric PfCdc48 is shown as cartoon. Different domains are marked and coloured: N-terminal domain (green); ATPase domain 1 (D1, magenta); ATPase domain 2 (D2, yellow). Loop between domain N and D1 (L1, dark blue), loop between domain D1 and D2 (L2, blue). Side chain of Cys98 is shown by spheres (orange). Figure was created with ChimeraX [56]. B) Multiple sequence alignment of *Plasmodium falciparum* PfCdc48 (Acc. N. XP_966179.2) with *Homo sapiens* Cdc48 isoform 1 (HsCDC48_1; NP_009057.1) and isoform 2 (HsCDC48_1; NP_001341856.1). Sequence are numbered according to PfCdc48. Different domains are coloured: N-terminal domain (green); ATPase domain 1 (D1, magenta); ATPase domain 2 (D2, yellow). Cys98 is highlighted (red) and

marked by asterisk. Gaps are indicated by dashes. Conserved residues in the alignment are indicated by dots.

A

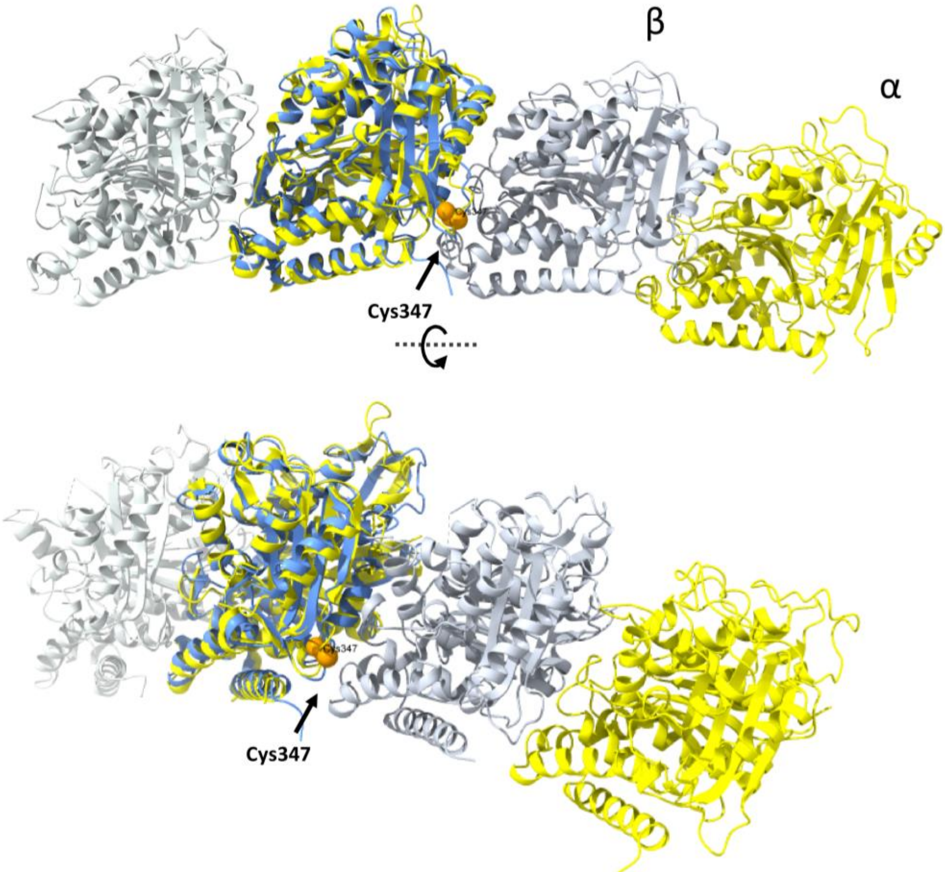

B

```
            1                                                                                                    100
PfTUBalpha  MREVISIHVGQAGIQIGNACWELFCLEHGIQPDGQMPSDQVVAGGDDAFNTFFSETGAGKHVPRCVFVDLEPTVVDEVRTGTYRQLFHPEQLISGKEDAA
HsTUBalpha  ...C.........V.........Y...............KTIG....S................A.........I..................T......
GdTUBalpha  ...C..V.I....V.........Y.......H.......KTIG.....................A..............A.S...Y..............
 HsTUBbeta  ..IVH.QA..C.N...AKF..VISD....D.T.SYHG.SDLQL--ERI.VYYN.AAGN.Y...AIL.....GTM.S..S.PFG.I.R.DNFVF.QSG.G

            101                                                                                                  200
PfTUBalpha  NNFARGHYTIGKEIVDVCLDRVRKLADNCTGLQGFLMFNAVGGGTGSGLGCLLLERLAIDYGKKSKLNFCSWPSPQVSTAVVEPYNSVLSTHSLLEHTDV
HsTUBalpha  ..Y.........I.LV...I.....Q..R.....V.HSF.......FTS..M...SV........E.SIY.A.............I.T..TT...S.C
GdTUBalpha  ..Y............LV...........S......I.HSF.....A.F.S.F....SV...R....E.VVY....IA........TI.AA..M...S.C
 HsTUBbeta  ..W.K....E.A.L..SV..V...ESES.DC....QLTHSL.......M.T..ISKIREE.PDRIMNT.SVM...K..DT......AT..V.Q.V.N..E

            201                                                                                                  300
PfTUBalpha  AIMLDNEAIYDICKKNLDIERPTYTNLNRLIAQVISSLTASLRFDGALNVDVTEFQTNLVPYPRIHFMLSSYAPIISAEKAYHEQLSVSEITNSAFEPAS
HsTUBalpha  .F.V.........RR................G.IV..I.............L..........P.AT...V.............A....AC....N
GdTUBalpha  .F.V...M....RR.................C...I.............L..........P.C.......S.....K...A.L...V....N
 HsTUBbeta  TYSI....L....FRT.KLTT...GD..H.VSATM.GV.TC...P.Q..A.LRKLAV.M..F..L..FMPGF..LT.RGSQQYRA.T.P.L.QQM.DSKN

            301                                                                                                  400
PfTUBalpha  MMAKCDPRHGKYMACCLMYRGDVVPKDVNAAVATIKTKRSIQFVDWCPTGFKCGINYQPPTVVPGGDLAKVMRAVCMISNSTAIAEVFSRMDQKFDLMYA
HsTUBalpha  Q.V....G.........L...........I.......T............V.................Q.....L..T.....AWA.L.H.......
GdTUBalpha  ..V............M...........I.V.....T...........V.........I........Q.SCL....T......W....H.......
 HsTUBbeta  ...A......R.LTVAAIF..RMSM.E.DEQMLNVQN.N.SY..E.I.NNV.TAVCDI..R--------GLKMSATF.G.....Q.L.K.ISEQ.TA.FR

            401                                                    455
PfTUBalpha  KRAFVHWYVGEGMEEGEFSEAREDLAALEKDYEEVGIESN-D-GEGEDEGYE
HsTUBalpha  ........................M...........VH.V-E-....E..E.Y
GdTUBalpha  ..................................I.A.TLG.-.....MEEDDAY
 HsTUBbeta  RK..L...T....D.M..T..ESNMND.VSE.QQYQDATADEQ..F.E.EG.DEA
```

Figure S9. Localization of 1-modified Cys98 in the PfTub-α2 modelled structure. A) Homology model of PfTub-α2 (blue) was obtained using SwissModel (see Materials and Methods), superimposed with Bos taurus α-tubulin in the α-tubulin (yellow)/β-tubulin (gray) oligomer structure (PDB ID: 4O2B), and visualized as cartoon. Tubulin subunits are indicated. Side chain of Cys347 is shown by spheres (orange). Figure was created with ChimeraX [56]. B) Multiple sequence alignment of Plasmodium

falciparum PfTub-α2 (PfalphaTUB; Acc. N. Q8IFP3) with Homo sapiens α-tubulin (HsalphaTUB; gi|37492) and β-tubulin (HsbetaTUB; CAA56071.1), and *Giardia duodenalis* α-tubulin (GdalphaTUB; GL50803_103676) Sequence are numbered according to PfTub-α2. Residues at the interface between α-tubulin β-tubulin are red, residues that bind GTP/GDP are in light blue [39]. Cys347 is highlighted (red) and marked by asterisk. Gaps are indicated by dashes. Conserved residues in the alignment are indicated by dots.
